# Toxicological assessment of novel Anti-COVID traditional Chinese medicine formulae NRICM101 and NRICM102: a comprehensive study on safety and genotoxicity

**DOI:** 10.3389/fphar.2025.1596369

**Published:** 2025-09-17

**Authors:** Chun-Tang Chiou, Chao-Lin Chang, Yu-Hwei Tseng, Geng-You Liao, Jiunn-Wang Liao, Yuh-Chiang Shen, Wen-Chi Wei, Keng-Chang Tsai, Yu-Ching Huang, Wen-Chiung Chang, Wen-Fei Chiou, Chia-Ching Liaw, Yi-Chang Su

**Affiliations:** ^1^ National Research Institute of Chinese Medicine, Ministry of Health and Welfare, Taipei, Taiwan; ^2^ Product and Process Research Center, Food Industry Research and Development Institute, Hsinchu, Taiwan; ^3^ Department of Public Health, National Cheng Kung University, Tainan, Taiwan; ^4^ Institute of Physiology, School of Medicine, National Yang Ming Chiao Tung University, Taipei, Taiwan; ^5^ Graduate Institute of Veterinary Pathobiology, National Chung Hsing University, Taichung, Taiwan; ^6^ Ph.D. Program in Medical Biotechnology, College of Medical Science and Technology, Taipei Medical University, Taipei, Taiwan; ^7^ Department of Biochemical Science and Technology, National Chiayi University, Chiayi, Taiwan; ^8^ Graduate Institute of Natural Products, Kaohsiung Medical University, Kaohsiung, Taiwan; ^9^ Department of Pharmacy, School of Pharmaceutical Sciences, National Yang Ming Chiao Tung University, Taipei, Taiwan; ^10^ School of Chinese Medicine, College of Medicine, National Yang Ming Chiao Tung University, Taipei, Taiwan

**Keywords:** NRICM101, NRICM102, novel TCM, COVID-19, safety, genotoxicity

## Abstract

Although the first outbreak of COVID-19 occurred in 2019, the virus continues to circulate globally, even years later. In Taiwan, the novel traditional Chinese medicine formulas, NRICM101 and NRICM102, have been extensively used to treat COVID-19, with Chinese medicine practitioners frequently prescribing them to manage the disease. According to data from the Taiwan Centers for Disease Control, approximately 22% of COVID-19 patients opted for NRICMs’ treatments between 2021 and 2022. Despite the widespread use and reported effectiveness of these treatments, it is critical to evaluate the potential risks associated with their prolonged or frequent use. In this study, we conducted a comprehensive toxicological assessment of NRICM101 and NRICM102. Acute oral toxicity was evaluated by administering a single 5 g/kg bw dose to ICR mice and SD rats. No mortality, sex-related differences, or clinical signs of toxicity were observed. Subchronic toxicity was assessed through a 28-day repeated oral administration study with doses of 1.6, 3.1, and 4.8 g/kg bw per day of NRICM101 or 102, which showed no treatment-related deaths or organ pathology. While some hematological changes were noted, they were generally within physiological ranges and showed no consistent dose-dependent trends. Genotoxicity was assessed using three standard assays. The Ames test revealed no mutagenic activity. The *in vitro* mouse lymphoma assay showed genotoxicity only at the highest concentration (5.0 mg/mL) and only in the absence of S9 metabolic activation, suggesting a context-dependent response possibly linked to direct-acting or cytotoxic effects at excessive doses. In contrast, the *in vivo* micronucleus assay, which reflects systemic genotoxicity under physiologically relevant conditions, showed negative results. Together, these findings indicate that NRICM101 and NRICM102 are not associated with acute or subchronic toxicity at clinically relevant doses and durations, and they present a low genotoxic risk under standard conditions of use. Nonetheless, further long-term and pharmacokinetic studies are warranted to fully characterize their safety profiles, particularly with high-dose or prolonged administration.

## 1 Introduction

The COVID-19 pandemic has claimed millions of lives worldwide in the past 3 years, and new cases continue to be reported. People with underlying conditions still face a higher risk of mortality and recurrent infections when repeated mutations evolve into strains with more severity. Existing immunomodulatory and antiviral therapies, as well as vaccines are imperfect, and their availability remains a challenge (Usher, 2022). The phenomenon is accompanied by the increasing use of traditional medicine. NRICM101 and NRICM102 are traditional Chinese medicine (TCM) formulae composed of multiple plants and are recommended as integrated therapeutic options for COVID-19 (Lai et al., 2023). NRICM101 was granted an emergency use authorization in Taiwan and is used in more than 50 countries worldwide, and NRICM102 was administered during a serious outbreak in mid-2021. The “Traditional Chinese Medicine Clinical Guideline for COVID-19” (TCM-CG) suggested integrated treatment of mild-to-moderate and severe-to-critical cases with TCM formulae NRICM101 and NRICM102, respectively. Both formulae comprise heat-clearing and detoxifying botanical drugs, with NRICM102 replacing some of the ingredients with others that enhance cardiovascular functions for severely ill patients (Su et al., 2022; Ho et al., 2023). Multicenter studies suggested that NRICM101 and NRICM102 improved symptoms of COVID-19 patients and significantly lowered the risk of death (Tsai et al., 2021; Tseng et al., 2022). Furthermore, the antiviral, anti-inflammatory, and immunomodulatory characteristics and mechanisms of the two TCM botanical drugs for inhibiting viral infection and replication and lung injury were demonstrated in pharmacological investigations (Tsai et al., 2021; Tseng et al., 2022; Wei et al., 2022; Cheng et al., 2022; Wei et al., 2023; Chang et al., 2024).

Traditional medicines such as NRICM101 and NRICM102, are generally considered to be effective, given their rising popularity at a global level. The association between use and adverse reactions is little researched. In particular, concerns persist regarding the lack of standardized quality control and comprehensive scientific evidence supporting the safety of many herbal preparations (Neergheen-Bhujun 2013; Tseng and Chang, 2019). Moreover, individual herbs can be well studied for their active properties; however, TCM preparations often comprise complex active ingredients whose synergism, antagonism, or toxicity potentiation warrant careful examination.

While individual ingredients of NRICM101 and 102 are classified as dietary supplements in many countries, including the United States, the safety aspects of their combined effects as a brand-new prescription remain unclear. Thus, addressing the issues regarding the association between plant metabolites and liver and kidney problems, the nervous system, cardiovascular system, and mutagenicity, among others, is necessary. To this end, in this study, we aimed to fill the knowledge gap by evaluating the safety of NRICM101 and 102, including genotoxicity using *in vitro* bacterial reverse mutation assay (Ames test), mouse lymphoma assay (MLA), micronucleus test, and single-dose acute and 28-day sub-acute toxicity tests in rats and mice. The safety and toxicity evaluations are expected to inform clinical practice and monitoring for potential adverse effects, in addition to characterizing the toxicological profile of NRICM101 and 102.

## 2 Materials and methods

### 2.1 Chemicals

The following chemicals were obtained from specified sources: 4-nitro-*O*-phenylenediamine (NPD, CAS No.: 99-56-9, Purity: 98%), sodium azide (SA, CAS No.: 26628-22-8), mitomycin c (MMC), benzo[α]pyrene (BP, CAS No.: 50-32-8), 2-aminoanthracene (2-AA, CAS No.: 613-13-8, Purity: 96%), Ethyl methanesulfonate (EMS, CAS No.: 62-50-0), *N*-(2-Fluorenyl) acetamide (2AAF, CAS No.: 53-96-3), Trifluorothymidine (TFT, CAS No.: 70–00–8), and Cyclophosphamide (CAS No.: 50-18-0) were purchased from Sigma (St. Louis, MO, USA). Additionally, 2-aminofluorene (2-AF, CAS No.: 153-78-6) was procured from Alfa Aesar (Haverhill, MA, USA), and S9, post-mitochondrial supernatant, was sourced from Moltox Company (Boone, NC, USA).

### 2.2 Formula and preparation of NRICM101 and 102

NRICM101 formula comprised baked Liquorice Root (7.50 g), Heartleaf Houttuynia (18.75 g), Indigowoad Root (18.75 g), Magnolia Bark (11.25 g), Peppermint Herb (11.25 g), Mulberry Leaf (11.25 g), Fineleaf Nepeta (11.25 g), Saposhnikovia Root (7.50 g), Scutellaria Root (18.75 g), and Mongolian Snakegourd Fruit (18.75 g), while the NRICM102 formula contains baked Liquorice Root (7.50 g), Heartleaf Houttuynia (18.75 g), Scutellaria Root (18.75 g), Magnolia Bark (11.25 g), Mongolian Snakegourd Fruit (18.75 g), Fragrant Solomonseal Rhizome (11.25 g), Indian Bread (18.75 g), Pinellia Tuber (11.25 g), Oriental Wormwood (18.75 g), and Prepared Common Monkshood Daughter Root (7.50 g). All botanical species referenced in NRICM101 and NRICM102 have been taxonomically validated by Dr. Chia-Ching Liaw (National Research Institute of Chinese Medicine, Ministry of Health and Welfare; Taipei 112, Taiwan), a qualified expert in pharmacognosy. Corresponding voucher specimens have been deposited at the Herbarium of the NRICM (MOHW, Taiwan). The decoctions of NRICM101 and 102 were further dried by cryodesiccation to obtain the extract powder. The chemical profiles and extract compositions of NRICM101 and 102 have been rigorously analyzed in our previous studies (Tsai et al., 2021; Wei et al., 2022).

The tested doses of NRICM101 or 102 were determined based on the recommended clinical dosage of 15 g of powder per day provided by the National Research Institute of Chinese Medicine (NRICM), MOWH. These doses were then normalized according to body surface area using allometric scaling (Chaturvedi et al., 2001). The calculation [15 g/60 kg × 0.625 = 1.6 g/kg/day] yielded a dose of 1.6 g per kg per day. For the 28-day repeated dose oral toxicity study, the tested doses were designed to correspond to 1-, 2-, and 3-fold of the human recommended doses. Specifically, the doses of 1.6 g per kg of body weight (low dose), 3.2 g per kg of body weight (middle dose), and 4.8 g per kg of body weight (high dose) were selected to represent the 1-, 2-, and 3-fold multiples of the recommended human doses, respectively.

### 2.3 Animals

All animal experiments received approval from the Institutional Animal Care and Use Committee (IACUC) and were conducted in accordance with the guidelines of “Good Laboratory Practice for Non-clinical Laboratory Studies” and “Healthy Food Safety Assessment” as prepared by the Ministry of Health and Welfare in Taiwan. For the acute oral toxicity test, a total of twenty animals were used, consisting of ten Institute for Cancer Research (ICR) mice (5 males and 5 females, aged 6–8 weeks) and ten Sprague-Dawley (SD) rats (5 males and 5 females, aged 5–6 weeks), all procured from BioLASCO Taiwan Co. They were randomly housed in polypropylene cages, with two animals per cage during the tests. For the repeated oral toxicity test, 80 SD rats (40 female and 40 male rats) obtained from BioLASCO Taiwan Co. were also randomly housed in polypropylene cages, with two animals per cage during the tests. The animals were maintained in conditions with a 12 h light/12 h dark cycle, a temperature range of 20 °C–24 °C, and a relative humidity range of 50%–70%. They had *ad libitum* access to a standard rodent diet (1326N, TPF; Altromin) and purified water. They were allowed to acclimate to their surroundings for 1–2 weeks before the start of the studies. The animal groupings for each toxicological assay are summarized below (Table 1).

**TABLE 1:** Summary of animal grouping for toxicity studies.

Testing	Animal	Grouping	Dosage	Sample size (n)
Acute Oral Toxicity (14 days)	ICR Mouse	Control	Sterile water	5 males +5 females
		NRICM101	5 g/kg bw (single dose)	5 males +5 females
		NRICM102	5 g/kg bw (single dose)	5 males +5 females
	SD Rat	Control	Sterile water	5 males +5 females
		NRICM101	5 g/kg bw (single dose)	5 males +5 females
		NRICM102	5 g/kg bw (single dose)	5 males +5 females
Subchronic Oral Toxicity (28 days)	SD Rat	Control	10 mL/kg bw/day (sterile water)	5 males +5 females
		Low	1.6 g/kg bw/day	5 males +5 females
		Middle	3.2 g/kg bw/day	5 males +5 females
		High	4.8 g/kg bw/day	5 males +5 females
Micronucleus Assay	ICR Mouse	Negative Control	Sterile water	5 males
		Positive Control	Cyclophosphamide (0.1 g/kg bw)	5 males
		Low	2.25 g/kg bw/day	5 males
		Middle	4.50 g/kg bw/day	5 males
		High	6.75 g/kg bw/day	5 males +5 females

### 2.4 Acute oral toxicity study: single-dose 14-day toxicity evaluation in rats and mice

For the acute oral toxicity study, ICR mice were used in accordance with the guidance of the Organization for Economic Cooperation and Development (OECD) No. 423 (OECD, 2002). Additionally, SD rats were included to enhance the robustness of the evaluation, enabling a more comprehensive assessment of the potential toxic effects of NRICM101 and 102. ICR mice were divided into four groups, each consisting of five males and five females. For NRICM101 and NRICM102, separate control groups (vehicle only) and treatment groups (5 g/kg bw) were established. SD rats were categorized into three groups for treatment with either NRICM101 and 102. Each group of SD rats comprised five individuals of each sex, including both control groups and those administered 5 g/kg body weight of NRICM101 or 102. The selection of 5 g/kg body weight for the acute oral toxicity test was based on the OECD #423 (OECD, 2002), which recommends testing within a range of 2–5 g/kg BW. The upper limit of this range was selected to thoroughly evaluate the acute safety profile under the most rigorous conditions. A single dose of 5 g/kg bw of NRICM101 or 102 was administered via gavage, with the negative control group receiving an equal volume of sterile water. The animals were weighed weekly, and their behavior, clinical signs, and mortality were monitored twice daily for 14 days. Following the 14-day acute oral toxicity testing period, the animals were euthanized, and serum samples were collected and analyzed for aspartate aminotransferase (AST), alanine aminotransferase (ALT), blood urea nitrogen (BUN), and creatinine (CREA). Data were expressed as mean ± standard deviation. One-way ANOVA followed by Dunnett’s *post hoc* test was used for multiple comparisons. A significance level of *P < 0.05 was considered statistically significant compared to the vehicle control.

### 2.5 Subchronic oral toxicity study: repeated-dose 28-day toxicity evaluation in rats

The 28-day repeated-dose oral toxicity study was performed in accordance with OECD #407: Repeated Dose 28-day Oral Toxicity Study in Rodents, to evaluate the systemic toxicity of NRICM formulations following subchronic oral exposure. Forty rats (comprising an equal number of females and males) were distributed into four groups: control (administered 10 mL of sterile water/kg bw/day); low dose (1.6 g/kg bw/day); middle dose (3.2 g/kg bw/day); and high dose (4.8 g/kg bw/day), with five female and five male rats in each group. Throughout the 28-day study period, all animals and food consumption were weighed weekly, and their behaviors, clinical signs, and mortality were monitored daily. Additionally, their body functions were assessed weekly through detailed examinations, including responses to stimuli, muscle strength, salivation, secretions, and respiratory sounds. At the end of the 28-day repeated administration, the animals were euthanized for a comprehensive evaluation, including clinical observations, hematology, serum biochemistry examinations, urinalysis, ophthalmological assessments, and pathological examinations. The vital organs were weighed, including the thymus, heart, spleen, kidney (paired organs were weighed together), adrenal gland, ovary, and brain. The tissues, including the adrenal gland, brain, heart, kidney, liver, lung, spleen, thymus, epididymis, testis, ovary, and oviduct, were fixed in 10% buffered formalin, stained with hematoxylin and eosin, and subjected to examination using light microscopy. This staining and subsequent microscopic assessment were performed on all tissues derived from both the control group and the high-dose NRICM101 or 102 groups.

Blood samples were subjected to hematological and serum biochemistry analysis with evaluation of following indices: RBC (red blood cell), HGB (hemoglobin), HCT (hematocrit), MCV (mean corpuscular volume), MCH (mean corpuscular hemoglobin), MCHC (mean corpuscular hemoglobin concentration), PLT (platelet), PT (prothrombin time), APTT (activated partial thromboplastin time), INR (international normalized ratio), NEU (neutrophil), Lym (lymphocyte), MONO (monocyte), EOS (eosinophil), BASO (basophil), ALB (albumin), AST (aspartate aminotransferase), ALT (alanine aminotransferase), GGT (γ-glutamyl transferase), T-BIL (total bilirubin), T-PROT (total protein), GLU (glucose), BUN CREA (creatinine), CHOL (cholesterol), HDL (high density lipoprotein), TG (triglyceride), P^3-^ (inorganic phosphorus), Na^+^ (sodium), K^+^ (potassium), Cl^−^ (chloride), and Ca^2+^ (calcium).

Urinalysis parameters included specific gravity, pH value, glucose levels, protein levels, bilirubin levels, urobilinogen levels, occult blood, ketone levels, nitrite levels, leukocyte count, urine volume over 6 h, and sediment composition, including white blood cells, RBC, casts, crystals (Cry), bacteria (Bac), squamous epithelial cells (NEC), and spermatozoa. The parameters of specific gravity, pH, leukocyte count, and urine volume (6 h) were quantitatively expressed, whereas some parameters listed in Table SX (Supplementary Material) were semi-quantitatively rated on a scale of “-”, “±“, “+“, “++“, “+++“, “++++“, corresponding to their levels. Data were expressed as mean ± standard deviation. One-way ANOVA followed by Dunnett’s *post hoc* test was used for multiple comparisons. A significance level of *P < 0.05 was considered statistically significant compared to the vehicle control.

### 2.6 Genotoxicity assessment 1: Ames bacterial reverse mutation test

The Ames test was performed in accordance with a previous study (Maron and Ames, 1983) and guidance of the OECD Guideline for the testing of chemicals #471: Bacterial reverse mutation test (OECD, 1997a). The test histidine-requiring strains of *Salmonella Typhimurium* used in the Ames test were TA97a, TA98, TA100, TA102, and TA1535, purchased from Molecular Toxicology Inc (Boone, NC, USA). Briefly, 100 μL of either NRICM101 or 102, along with 0.1 mL of each strain suspension, were introduced into 2 mL of molten top agar containing 0.5 mM histidine/biotin. The mixture was poured onto minimal glucose agar plates and incubated at 37 °C for 48 h, after which the number of revertant colonies was manually counted. For assays with metabolic activation, 0.5 mL of S9 mix was added, consisting of 5% S9 post-mitochondrial fraction, 4 mM β-NADP, 5 mM glucose-6-phosphate, 8 mM MgCl_2_, 8 mM KCl, and 100 mM sodium phosphate buffer. In the absence of metabolic activation, 0.5 mL of 0.2 M sodium phosphate buffer (pH 7.4) was used instead. NRICM101 and NRICM102 were prepared as 50 mg/mL stock solutions in sterile water, centrifuged to remove particulates, and filtered through a 0.22-µm membrane before use. Based on the pre-test results for cytotoxicity, 5 mg/plate of NRICM101 or 102 exhibited no substantial cytotoxic effects on TA97a, TA98, TA100, TA102, and TA1535 strains. Therefore, five doses (0.3125, 0.625, 1.25, 2.5, and 5.0 mg/plate) were selected for both the presence (+S9) and absence (-S9) of metabolic activation. The negative and positive controls were prepared under the same conditions. Sterile water served as the negative control. For the -S9 condition, the positive controls included 4-nitro-o-phenylenediamine (NPD) for TA97a and TA98, sodium azide (SA, 0.4 µg/plate) for TA100 and TA1535, and mitomycin C (MMC, 0.5 µg/plate) for TA102. For the +S9 condition, the positive controls included 2-aminofluorene (2-AF, 4.0 µg/plate) for TA97a and TA100, benzo[a]pyrene (BP, 4.0 µg/plate) for TA98, and 2-aminoanthracene (2-AA, 4.0 µg/plate) for TA102 and TA1535. All treatments were conducted in triplicate for each dose level and control. Plates were incubated for 48 h before evaluating revertant colony counts to assess mutagenic potential. Data were expressed as mean ± standard deviation. A positive mutagenic response was indicated by the presence of revertant colonies exhibiting more than a 2-fold increase relative to the negative control in the TA97a, TA98, TA100, and TA102 strains, or a greater than 3-fold increase in TA1535. Conversely, if no such increases were observed in any of the tested strains, the results were deemed negative for mutagenic potential.

### 2.7 Genotoxicity assessment 2: mouse lymphoma thymidine kinase gene mutation assay (MLA)

The *in vitro* MLA assay was performed in accordance with the OECD Guidelines for the Testing of Chemicals #490: *In Vitro* Mammalian Cell Gene Mutation Tests Using the Thymidine Kinase Gene (OECD, 1997b). The L5178Y TK^+/−^ mouse lymphoma cell line used in the assay was obtained from the Bioresource Collection and Research Center (Hsinchu, Taiwan). Prior to testing, the cells were pre-cultured in medium containing thymidine, hypoxanthine, methotrexate, and glycine for 24 h to eliminate pre-existing TK^−/−^ mutants (Clive and Spector, 1975). Subsequently, the cells were cultured for an additional 48 h in RPMI-1640 medium supplemented with 20% heat-inactivated horse serum (HI-HS) and 0.1% Pluronic F-68, at a density of 1 × 10^5^ cells/mL in T25 flasks. NRICM101 and NRICM102 were prepared as 50 mg/mL stock solutions in Dulbecco’s phosphate-buffered saline (D-PBS), vortexed for 5 min, and centrifuged at 2,000 rpm for 5 min to remove insoluble particles. The supernatants were then sterilized by filtration through a 0.22-μm membrane filter. Based on preliminary cytotoxicity testing, three test concentrations (1.25, 2.5, and 5.0 mg/mL) were selected for the MLA assay. These working solutions were freshly diluted from the 50 mg/mL stock using D-PBS. D-PBS was used as the negative control. Ethyl methanesulfonate (EMS, 0.32 mg/mL) and N-(2-fluorenyl)acetamide (2-AAF, 0.2 mg/mL) were used as positive controls for assays conducted without and with metabolic activation (S9), respectively. Following treatment, cells were cultured either at 10 cells/mL (2 cells/well in 96-well plates) in non-selective medium for cloning efficiency, or at 1 × 10^4^ cells/mL (2,000 cells/well) in selective medium containing trifluorothymidine (TFT; Sigma) to select for TK^−^ mutants. Both sets of cultures were incubated for 12 days at 37 °C in a humidified atmosphere with 5% CO_2_. After incubation, mutant colonies were manually counted, and colony size (small or large) was classified according to OECD Guideline No. 490. The following parameters were calculated: the number of empty wells divided by the total wells plated P(0), plating efficiency (P.E.%), and cloning efficiency (C.E.%). P(0) = number of empty wells/total wells plated; P.E.% = (-lnP(0)/number of cells per well, two cells per well/in non-selective medium)×100%; C.E.% = (-lnP(0)/number of cells per well, 2,000 cells per well/in selective medium)×100%. Subsequently, the mutation frequency (MF) was calculated using formula M.F. per survival = C.E.% in selective medium/P.E.% in non-selective medium. Data were expressed as mean ± standard deviation. An MF value exceeding a 2-fold increase compared to that observed in the negative control indicated an increase in mutagenicity.

### 2.8 Genotoxicity assessment 3: micronucleus assay in mouse peripheral blood

The micronuclei assay in the mouse peripheral blood was performed also in accordance with the guidelines of “Good Laboratory Practice for Non-clinical Laboratory Studies” and “Healthy Food Safety Assessment” and the recommendations of OECD guideline #474 (OECD, 1997b). For the micronuclei assay, Thirty-six ICR mice (comprising Thirty-one males and five females) were distributed into five groups: negative control (administered sterile water), positive control (cyclophosphamide, 0.1 g/kg bw), low dose (2.25 g/kg bw/day), middle dose (4.50 g/kg bw/day), and high dose (6.75 g/kg bw/day), with five male rats in each group and a female high dose group. The time points for assessing the micronucleus test in mouse peripheral blood, following the preliminary test of the high dose (6.75 g/kg bw/day) of NRICM101 or 102, were selected at 48 h after administration. The micronucleus test was performed on peripheral blood cells by cardiac puncture blood collection. At the end of the study, animals were sacrificed, and the peripheral blood was collected and mixed with heparin and subsequently fixed in methanol at −80 °C overnight. Then, samples were mixed with NaHCO_3_ buffer and centrifuged at 1,000 for 5 min. The blood cells in the pellet were re-suspended in combination with FITC-CD71 and RNase. After incubation, the samples were further stained with propidium iodide (PI) before subjected them to flow cytometry analysis (BD FACScalibur, BD Biosciences, US) and analyzed using Cell Quest software (BD Biosciences, US). The ratio of micronucleated reticulocytes to total reticulocytes was calculated by counting 5,000 reticulocytes per animal using a flow cytometer. Data were expressed as mean ± standard deviation. One-way ANOVA followed by Dunnett’s *post hoc* test was used for multiple comparisons. A significance level of *P < 0.05 was considered statistically significant compared to the vehicle control.

### 2.9 Statistical analysis

All data were expressed as mean ± standard deviation. Data from the acute and repeated oral toxicity studies (including body weight, absolute and relative organ weights, hematology, serum biochemistry, and urinalysis) as well as the *in vivo* micronucleus test, were analyzed using one-way ANOVA followed by Dunnett’s *post hoc* test for multiple comparisons (SPSS 9.0; SPSS Inc., Cary, NC). A significance level of *P < 0.05 was considered statistically significant compared to the vehicle control.

## 3 Results

### 3.1 Acute oral toxicity study of NRICM101/102 in mice and rats

**FIGURE 1:**
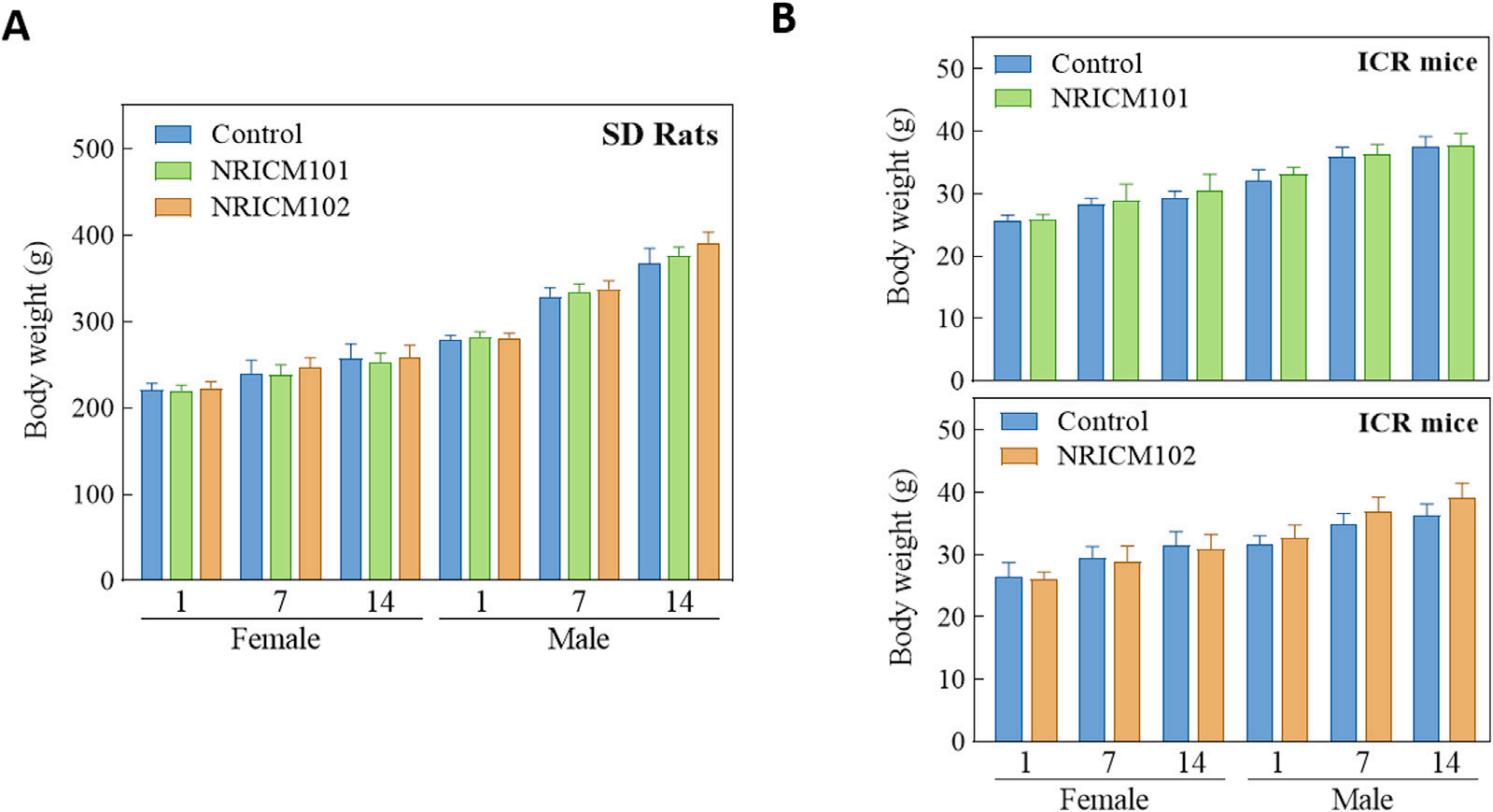
Effect of NRICM101 or 102 on body weight gain in the single doe acute oral toxicity test. Male and female SD rats **(A)** and ICR mice **(B)** were administered a single dose of NRICM101 or NRICM102 (5 g/kg bw) or an equal volume of sterile water (control). Body weight gain was monitored for 14 days post-administration. Data are presented as mean ± standard deviation (n = 5).

**FIGURE 2:**
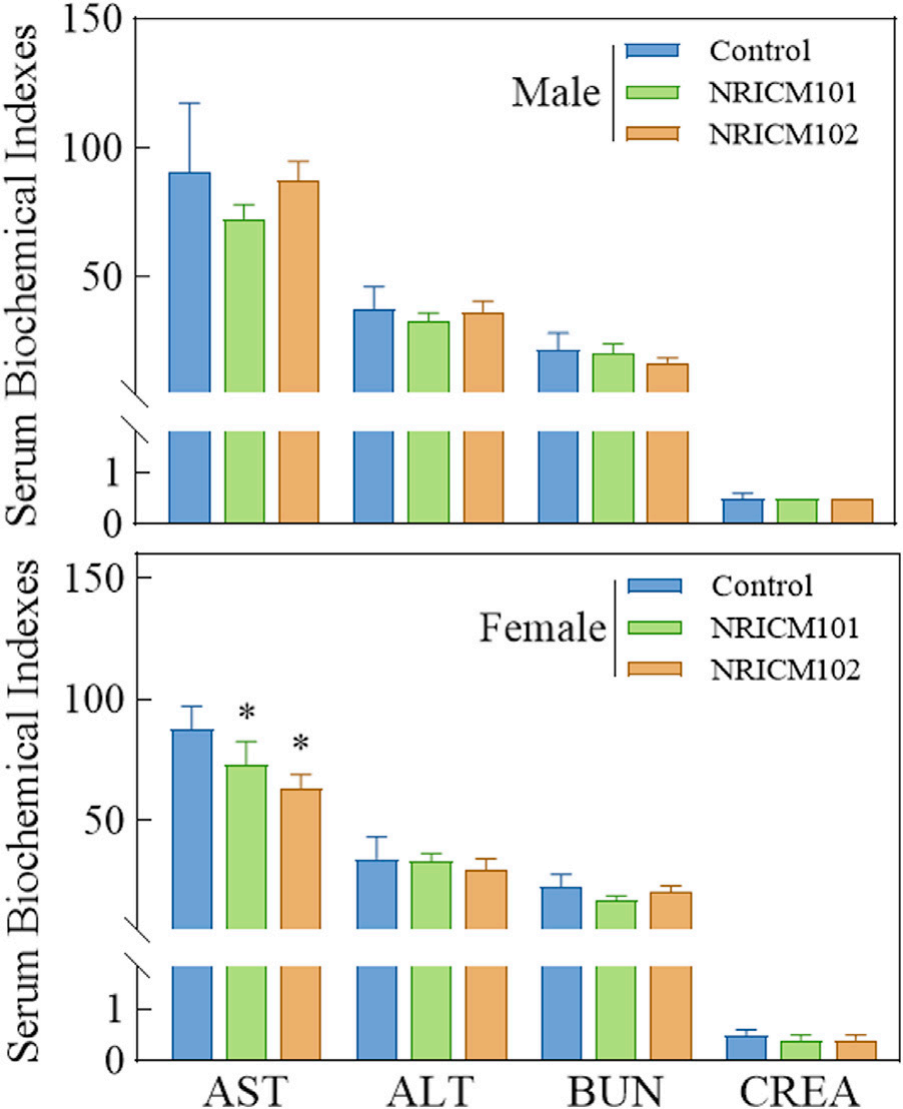
Effect of NRICM101 or 102 on serum biochemical parameters in the acute oral toxicity study in rats. Male and female rats (5 animals per group) were administered a single oral dose of NRICM101 or NRICM102 (5 g/kg bw) or an equal volume of sterile water (control). After 14 days, serum samples were collected prior to sacrifice and analyzed for aspartate aminotransferase (AST), alanine aminotransferase (ALT), blood urea nitrogen (BUN), and creatinine (CREA) levels. Data are presented as mean ± standard deviation (n = 5). *P < 0.05 vs. control group.

We assessed the potential toxicity of NRICM101 and 102 by administering a single 5 g/kg bw dose orally to ICR mice and SD rats. Over a 14-day observation period, we monitored behaviors, mortality, clinical signs, and body weights, and found no deaths, unusual behavior changes, abnormal clinical signs, or body weight variations in either group (Figure 1; Supplementary Table S1). Furthermore, liver and renal functions were evaluated through serum biochemistry analysis. In male SD rats treated with NRICM101 or 102, no significant changes were noted in alanine ALT, AST, BUN, or CREA levels when compared to the control group (Figure 2; Supplementary Table S2). In contrast, female rats treated with NRICM101 or 102 displayed no significant variations in ALT, BUN, and CREA levels but showed a lower AST level compared to the control group. Lastly, all animals were sacrificed and subjected to visual organ inspection, including the brain, spleen, liver, heart, lung, gut, kidney, testes, and ovaries. No clinical abnormalities related to the administration of NRICM101 and 102 were observed.

### 3.2 A 28-day repeated oral sub-acute toxicity study of NRICM101 and 102 in rats

#### 3.2.1 Clinical observation, body weight changes, and food consumption

**FIGURE 3:**
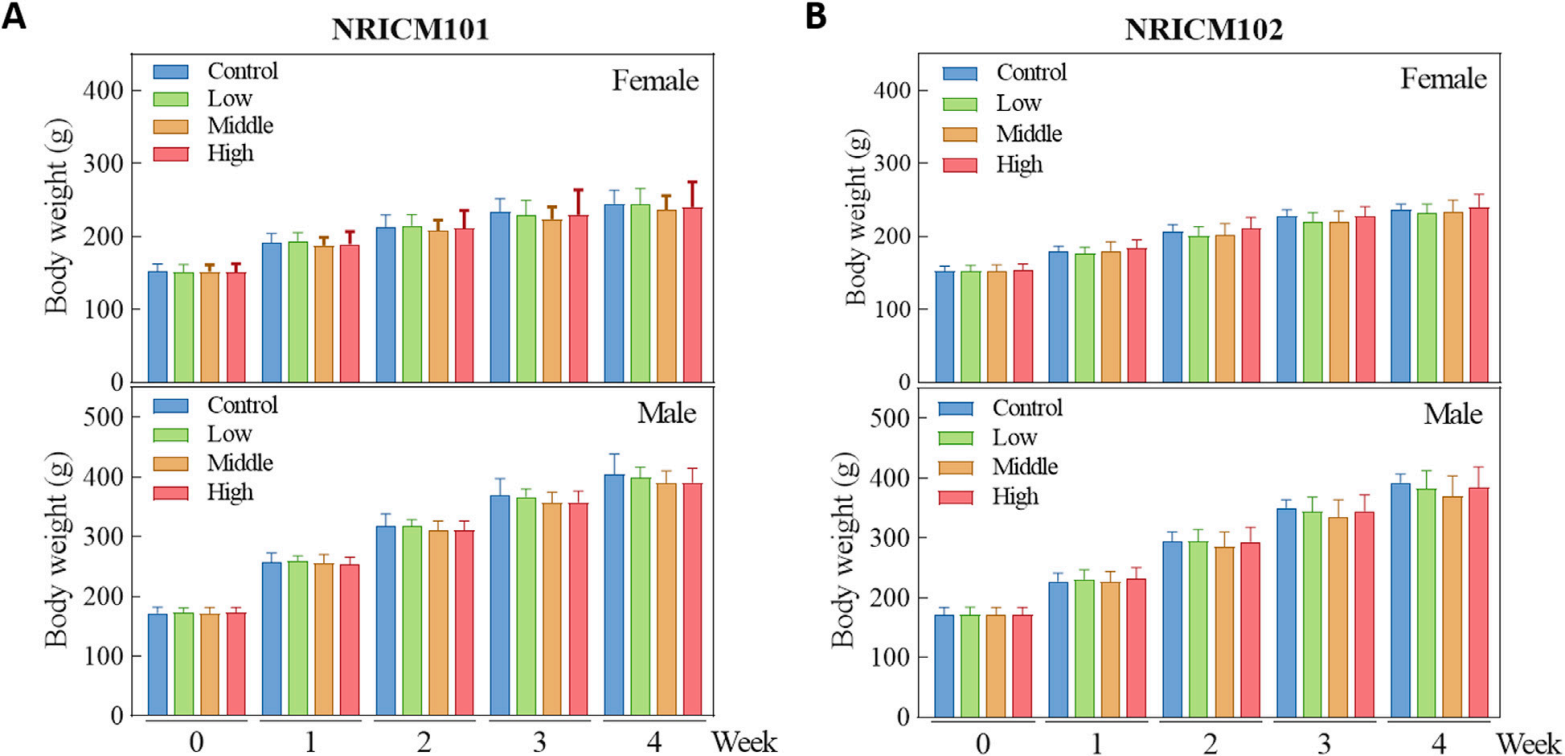
Effect of NRICM101 or NRICM102 on body weight in SD rats during a 28-day repeated oral toxicity study. Male and female SD rats were administered Male and female SD rats were administered NRICM101 **(A)** or NRICM102 **(B)** once daily for 28 days at low (1.6 g/kg bw/day), middle (3.2 g/kg bw/day), or high (4.8 g/kg bw/day) doses. Control animals received sterile water (10 mL/kg bw/day). Body weights were recorded weekly. Data are presented as mean ± standard deviation (n = 5).

**FIGURE 4:**
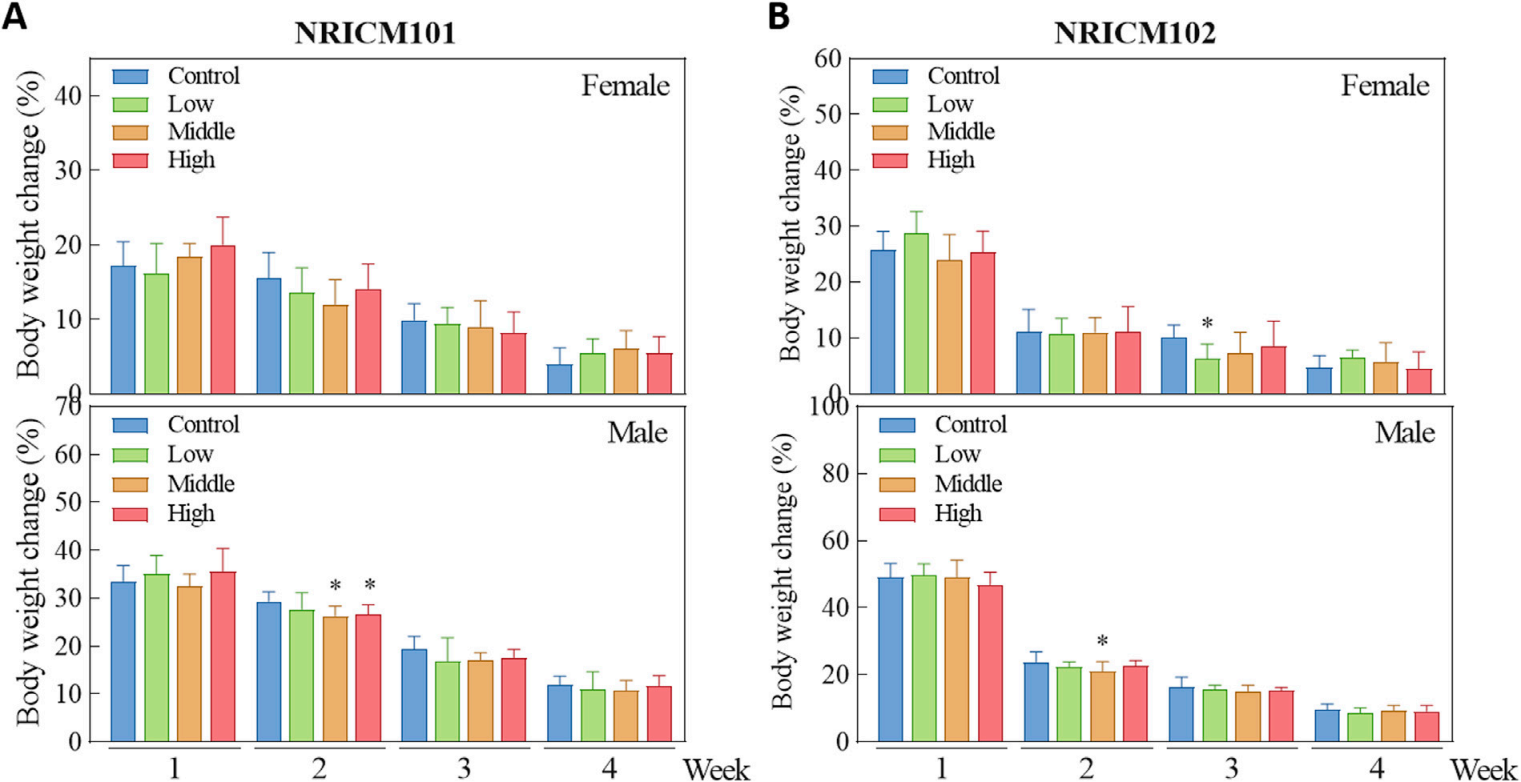
Effect of NRICM101 or 102 on body weight change in the repeated subacute oral toxicity. Animals were subjected to treatment as described in Figure 3. Animals were administered Animals were administered NRICM101 **(A)** or NRICM102 **(B)** once daily for 28 days at low (1.6 g/kg bw/day), middle (3.2 g/kg bw/day), or high (4.8 g/kg bw/day) doses. Control animals received an equal volume of sterile water (10 mL/kg bw/day). Weekly body weight changes were calculated by the following equation. *Body weight change* (%) = [BW of week #n-BW of week #(n-1)]/[BW of week#(n-1)]×100%. Data are presented as mean ± standard deviation (n = 10). *P < 0.05 vs. control group.

**FIGURE 5:**
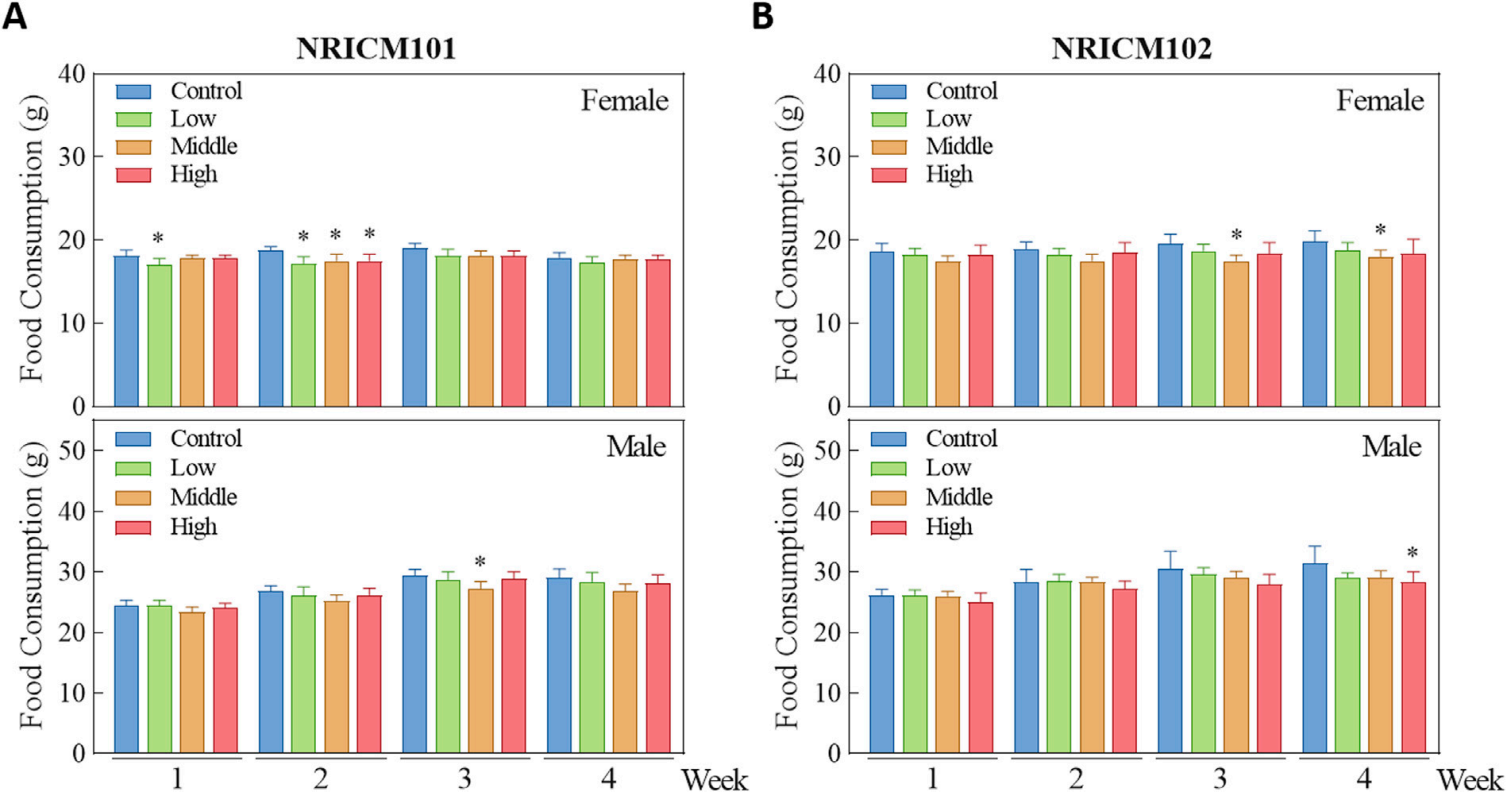
Effect of NRICM101 or NRICM102 on food consumption in SD rats during a 28-day repeated oral toxicity study. Animals were subjected to treatment as described in Figure 3. Animals were administered NRICM101 **(A)** or NRICM102 **(B)** once daily for 28 days at low (1.6 g/kg bw/day), middle (3.2 g/kg bw/day), or high (4.8 g/kg bw/day) doses. Control animals received an equal volume of sterile water (10 mL/kg bw/day). Food consumption was measured weekly throughout the study. Data are presented as mean ± standard deviation (n = 10). *P < 0.05 vs. control group.

We assessed sub-acute chronic toxicity by orally administering NRICM101 or 102 to SD rats at doses of 0 (vehicle control), 1.6 (low dose), 3.2 (mid dose), or 4.8 g/kg bw/day (high dose) for 28 days. Throughout the 28-day study, no abnormal clinical signs and deaths were noted. Regarding body weight (Figure 3; Supplementary Table S3), neither male nor female rats treated with any dose of NRICM101 or NRICM102 showed significant changes. For percent weight change (Figure 4; Supplementary Table S4), NRICM101-treated females showed no significant differences at any dose. In males, body weight change was slightly reduced during the second week in the mid- and high-dose groups, but normalized in subsequent weeks. For NRICM102, high-dose males and females showed no significant changes in weight. However, males in the mid-dose group exhibited slightly reduced body weight change in week 2, and females in the low-dose group showed a decrease in week 3. However, these fluctuations in both NRICM101 and 102 administration were rapidly normalized, with animals regaining weight at rates similar to the control group in subsequent weeks. Food consumption (Figure 5; Supplementary Table S5) showed notable reductions in some groups, but there was no consistent dose-dependence observed. In females, decreased food consumption occurred in the low-dose NRICM101 group during weeks 1 and 2, the middle-dose NRICM101 group during weeks 2 and 3, and the high-dose NRICM101 group during week 2. Among males, a decline in food consumption was observed in the middle-dose NRICM101 group during week 3. For NRICM102, females in the middle-dose group had reduced consumption in weeks 3 and 4, and males in the high-dose group in week 4. These sporadic changes were not considered treatment-related. Overall, no sustained or dose-dependent differences in body weight or food intake were observed.

#### 3.2.2 Analysis of relative changes of organ weights (ROW) during repeated sub-acute oral toxicity test

Following the 28-day repeated oral administration, we conducted examinations on all animals, including assessments of major organ appearances, ROWs, and clinical pathology analysis. No unusual appearances were noted in major organs such as the thymus, heart, spleen, liver, kidney, adrenal gland, ovary, testicle, and brain for rats subjected to either NRICM101 or 102. The assessment of external appearance and internal or structure of eyes also showed no difference compared to the control group. In NRICM101-treated rats, ROWs of major organs did not significantly differ from controls in either sex (Table 2). In NRICM102-treated rats, females in the mid-dose group and males in the low-dose group showed significantly increased adrenal gland ROWs compared to controls, while no differences were observed in the other dose groups.

**TABLE 2:** Effect of NRICM101 or 102 on relative organ weights (ROWs) in the repeated subacute oral toxicity test.

Organ	NRICM101				NRICM102			
	Control^a^	Low^b^	Middle	High	Control	Low	Middle	High
	Female				Female			
Thymus	0.20 ± 0.04	0.18 ± 0.03	0.18 ± 0.04	0.20 ± 0.03	0.18 ± 0.05	0.20 ± 0.04	0.18 ± 0.03	0.20 ± 0.06
Heart	0.37 ± 0.04	0.33 ± 0.03	0.35 ± 0.03	0.36 ± 0.04	0.34 ± 0.02	0.34 ± 0.03	0.35 ± 0.03	0.34 ± 0.03
Spleen	0.24 ± 0.06	0.21 ± 0.03	0.20 ± 0.03	0.21 ± 0.03	0.21 ± 0.02	0.21 ± 0.02	0.22 ± 0.03	0.20 ± 0.02
Liver	2.98 ± 0.17	2.99 ± 0.29	2.82 ± 0.20	2.89 ± 0.23	2.85 ± 0.24	3.10 ± 0.16	2.88 ± 0.12	2.85 ± 0.18
Kidney	0.73 ± 0.05	0.73 ± 0.05	0.74 ± 0.06	0.72 ± 0.06	0.69 ± 0.05	0.73 ± 0.04	0.72 ± 0.04	0.71 ± 0.02
Adrenal gland	0.03 ± 0.01	0.02 ± 0.00	0.03 ± 0.01	0.02 ± 0.00	0.02 ± 0.00	0.03 ± 0.01	0.03 ± 0.01*	0.02 ± 0.01
Ovary	0.06 ± 0.01	0.06 ± 0.01	0.06 ± 0.01	0.06 ± 0.01	0.05 ± 0.00	0.06 ± 0.02	0.06 ± 0.02	0.06 ± 0.01
Brain	0.84 ± 0.04	0.85 ± 0.05	0.86 ± 0.05	0.82 ± 0.07	0.82 ± 0.06	0.82 ± 0.07	0.83 ± 0.07	0.82 ± 0.12

	Male				Male			
Thymus	0.17 ± 0.04	0.16 ± 0.02	0.15 ± 0.02	0.15 ± 0.04	0.15 ± 0.05	0.15 ± 0.05	0.14 ± 0.04	0.14 ± 0.04
Heart	0.35 ± 0.03	0.42 ± 0.20	0.35 ± 0.04	0.36 ± 0.05	0.35 ± 0.03	0.35 ± 0.03	0.37 ± 0.05	0.37 ± 0.05
Spleen	0.18 ± 0.02	0.17 ± 0.02	0.18 ± 0.01	0.17 ± 0.02	0.18 ± 0.02	0.18 ± 0.02	0.18 ± 0.03	0.18 ± 0.03
Liver	3.19 ± 0.24	3.19 ± 0.20	3.17 ± 0.17	3.18 ± 0.20	3.07 ± 0.18	3.07 ± 0.18	3.01 ± 0.25	3.01 ± 0.25
Kidney	0.71 ± 0.05	0.76 ± 0.15	0.72 ± 0.04	0.75 ± 0.05	0.69 ± 0.03	0.69 ± 0.03	0.72 ± 0.03	0.72 ± 0.03
Adrenal gland	0.01 ± 0.00	0.01 ± 0.00	0.01 ± 0.00	0.01 ± 0.00	0.01 ± 0.00	0.02 ± 0.00*	0.02 ± 0.00	0.02 ± 0.00
Testes	0.86 ± 0.07	0.88 ± 0.09	0.89 ± 0.08	0.87 ± 0.11	0.81 ± 0.04	0.81 ± 0.04	0.81 ± 0.15	0.81 ± 0.15
Brain	0.55 ± 0.02	0.57 ± 0.04	0.59 ± 0.05	0.55 ± 0.04	0.54 ± 0.03	0.54 ± 0.03	0.55 ± 0.02	0.55 ± 0.02

Data were presented as means ± standard deviation (n = 10, per group for male and female rats, after 28-day repeated oral administration). *P < 0.05 vs. the control group.

^a^ Control: equal volume of sterile water.

^b^ NRICM101 or 102, Low: 1.6 g/kg bw/day; Middle: 3.2 g/kg bw/day; High: 4.8 g/kg bw/day.

#### 3.2.3 Hematological, serum biochemical, urine, and urinary sediment analysis

Hematological analysis showed that low-dose NRICM101 had no significant impact on hematological indices in both male and female rats (Table 3). Middle-dose NRICM101 resulted in significant increases in RBC (7.32 ± 0.36 × 10^6^/μL) and HCT (42.9% ± 2.1%) levels in female rats, along with a decrease in MCHC (33.9 ± 0.9 g/dL), compared to the RBC (6.77 ± 0.40 × 10^6^/μL), HCT (39.8% ± 2.3%), and MCHC (34.9 ± 0.8 g/dL) levels of the control group. No significant changes in other hematological parameters were observed. In male rats, administration of middle-dose NRICM101 did not lead to significant differences in hematological parameters. In high-dose NRICM101-treated females, RBC (7.32 ± 0.44 × 10^6^/μL), HCT (43.8% ± 2.5%), and HGB (15.0 ± 0.6 g/dL) levels were significantly elevated, while Ca^2+^ levels decreased (10.1 ± 0.2 mg/dL) compared to controls (RBC: 6.77 ± 0.40 × 10^6^/μL; HCT: 39.8% ± 2.3 %; HGB: 13.9 ± 0.7 g/dL; Ca^2+^: 10.5 ± 0.4 mg/dL). In high-dose males, PT (13.4 ± 2.2 s) and INR (1.2 ± 0.2) were significantly increased relative to controls (PT: 10.7 ± 0.4 s; INR: 1.0 ± 0.1), with no other notable changes. For NRICM102, only low-dose-treated males showed a significant reduction in PLT (1104 ± 132 × 10^3^/μL) compared to controls (1274 ± 155 × 10^3^/μL), with no significant alterations in other hematological parameters across all groups. Regarding urine and urinary sediments analysis, neither NRICM101 nor 102 administration affected urine or urinary sediments in either male or female rat groups at any dosage level (Tables 4, 5).

**TABLE 3:** Summary of hematology and serum biochemistry parameters.

Index	NRICM101				NRICM102			
	Control^a^	Low^b^	Middle	High	Control	Low	Middle	High
	Female/Male				Female/Male			
WBC (10^3^/mL)	7.7 ± 2.3/9.7 ± 1.9	5.8 ± 1.4/9.1 ± 1.7	6.9 ± 2.4/10.6 ± 2.2	6.0 ± 1.8/9.0 ± 2.3	6.5 ± 1.5/10.3 ± 1.8	6.5 ± 2.5/9.6 ± 1.9	7.4 ± 2.2/10.6 ± 1.5	5.1 ± 1.8/10.5 ± 3.3
RBC (10^6^/mL)	6.77 ± 0.40/7.62 ± 0.31	7.12 ± 0.38/7.69 ± 0.42	7.32 ± 0.36*/7.34 ± 0.94	7.32 ± 0.44*/7.62 ± 0.27	7.63 ± 0.3/8.43 ± 0.5	7.38 ± 0.3/8.77 ± 0.6	7.63 ± 0.6/8.39 ± 0.7	7.66 ± 0.3/8.32 ± 0.5
HGB (g/dL)	13.9 ± 0.7/15.3 ± 0.4	14.4 ± 0.5/15.4 ± 0.5	14.5 ± 0.9/15.0 ± 0.5	15.0 ± 0.6*/15.0 ± 0.4	15.3 ± 0.7/16.3 ± 1.0	14.8 ± 0.4/17.5 ± 1.6	14.9 ± 0.8/16.1 ± 1.3	14.9 ± 0.7/16.3 ± 1.2
HCT (%)	39.8 ± 2.3/46.8 ± 1.8	42.0 ± 1.7/47.2 ± 1.8	42.9 ± 2.1*/46.6 ± 1.1	43.8 ± 2.5*/46.3 ± 1.5	44.3 ± 1.8/50.0 ± 2.2	43.3 ± 1.6/52.6 ± 3.6	44.1 ± 2.7/50.2 ± 3.8	44.6 ± 2.1/50.3 ± 2.7
MCV (fL)	58.9 ± 1.4/61.4 ± 1.6	58.9 ± 1.5/61.5 ± 2.4	58.6 ± 1.4/61.0 ± 1.1	60.0 ± 1.2/60.8 ± 1.3	58.1 ± 1.4/59.4 ± 2.0	58.7 ± 1.0/60.0 ± 1.2	57.9 ± 1.1/59.9 ± 2.7	58.1 ± 1.4/60.5 ± 1.1
MCH (pg)	20.5 ± 0.8/20.0 ± 0.6	20.3 ± 0.6/20.1 ± 0.8	19.9 ± 0.8/19.7 ± 0.5	20.5 ± 0.7/19.7 ± 0.5	19.9 ± 0.7/19.4 ± 1.1	20.0 ± 0.6/19.9 ± 0.7	19.6 ± 0.7/19.3 ± 1.2	19.5 ± 0.5/19.6 ± 0.5
MCHC (g/dL)	34.9 ± 0.8/32.6 ± 0.8	34.4 ± 0.5/32.7 ± 0.9	33.9 ± 0.9*/32.2 ± 0.8	34.3 ± 1.0/32.5 ± 0.8	34.3 ± 1.0/32.6 ± 1.1	34.1 ± 0.8/33.2 ± 1.0	33.9 ± 0.8/32.1 ± 0.8	33.5 ± 0.8/32.3 ± 1.0
PLT (10^3^/mL)	1324 ± 175/1433 ± 176	1247 ± 169/1342 ± 79	1324 ± 208/1321 ± 146	1269 ± 150/1322 ± 185	1266 ± 152/1274 ± 155	1180 ± 170/1104 ± 132*	1253 ± 162/1267 ± 132	1173 ± 110/1154 ± 121
PT (sec)	10.9 ± 0.7/10.7 ± 0.4	10.5 ± 0.3/11.7 ± 1.2	10.8 ± 0.7/11.7 ± 1.1	10.4 ± 0.5/13.4 ± 2.2*	11.1 ± 0.5/12.3 ± 2.1	11.1 ± 0.5/12.2 ± 1.0	11.2 ± 0.4/13.2 ± 2.8	11.2 ± 0.2/12.7 ± 1.5
APTT (sec)	33.0 ± 8.1/29.3 ± 9.5	31.4 ± 8.7/32.7 ± 11.4	30.1 ± 6.8/27.4 ± 10.3	33.8 ± 9.2/28.9 ± 8.4	27.1 ± 7.7/31.4 ± 7.9	31.9 ± 9.8/36.0 ± 8.1	24.8 ± 4.9/35.8 ± 8.3	29.8 ± 9.4/27.6 ± 9.6
INR	0.96 ± 0.06/0.9 ± 0.0	0.93 ± 0.03/1.0 ± 0.1	0.95 ± 0.06/1.0 ± 0.1	0.92 ± 0.05/1.2 ± 0.2*	0.99 ± 0.05/1.09 ± 0.18	0.99 ± 0.04/1.09 ± 0.08	1.00 ± 0.04/1.17 ± 0.24	1.00 ± 0.02/1.13 ± 0.13
NEU (%)	15.2 ± 4.4/12.4 ± 2.8	14.6 ± 7.3/11.9 ± 3.4	14.7 ± 5.8/12.1 ± 2.5	13.2 ± 3.0/11.7 ± 3.4	13.5 ± 3.6/10.1 ± 2.0	11.5 ± 2.2/9.5 ± 2.3	12.5 ± 3.7/10.1 ± 2.5	14.2 ± 5.1/10.8 ± 3.0
LYM (%)	80.4 ± 5.5/82.3 ± 4.3	81.7 ± 7.4/82.9 ± 5.4	81.2 ± 6.3/83.8 ± 2.9	82.9 ± 3.2/84.1 ± 4.8	80.5 ± 5.6/86.2 ± 3.0	83.1 ± 3.4/86.9 ± 3.5	81.4 ± 5.4/87.3 ± 2.9	79.8 ± 5.3/86.5 ± 3.7
MONO (%)	2.7 ± 1.1/4.4 ± 2.1	2.0 ± 0.6/4.0 ± 2.4	2.7 ± 0.9/3.3 ± 1.6	2.5 ± 0.9/3.1 ± 1.7	4.5 ± 2.3/2.5 ± 1.4	3.7 ± 1.5/2.4 ± 1.8	4.0 ± 1.8/1.6 ± 1.0	4.3 ± 2.0/1.5 ± 1.3
EOS (%)	1.6 ± 0.8/0.9 ± 0.3	1.6 ± 0.5/1.1 ± 0.5	1.4 ± 0.6/0.7 ± 0.2	1.3 ± 0.5/1.0 ± 0.4	1.3 ± 0.5/1.2 ± 0.4	1.7 ± 0.6/1.1 ± 0.3	2.0 ± 0.7/1.0 ± 0.3	1.6 ± 0.8/1.1 ± 0.3
BASO (%)	0.1 ± 0.1/0.1 ± 0.0	0.1 ± 0.1/0.1 ± 0.1	0.1 ± 0.1/0.1 ± 0.0	0.1 ± 0.1/0.1 ± 0.1	0.1 ± 0.1/0.1 ± 0.1	0.0 ± 0.0/0.1 ± 0.0	0.1 ± 0.1/0.1 ± 0.0	0.1 ± 0.1/0.1 ± 0.0
ALB (g/dL)	3.5 ± 0.3/3.1 ± 0.2	3.2 ± 1.0/3.0 ± 0.1	3.4 ± 0.2/2.9 ± 0.2	3.6 ± 0.2/2.9 ± 0.1	3.4 ± 0.2/3.2 ± 0.1	3.6 ± 0.3/3.1 ± 0.1	3.4 ± 0.2/3.2 ± 0.1	3.4 ± 0.2/3.1 ± 0.1
ALP (U/l)	101 ± 26/184 ± 39	113 ± 40/174 ± 34	103 ± 22/176 ± 34	115 ± 25/186 ± 22	131 ± 38/196 ± 44	126 ± 39/221 ± 48	110 ± 21/196 ± 44	108 ± 27/221 ± 48
ALT (U/l)	33 ± 7/35 ± 8	29 ± 12/32 ± 3	27 ± 5/32 ± 5	26 ± 4/32 ± 4	31 ± 7/33 ± 5	31 ± 9/40 ± 9	29 ± 5/32 ± 6	26 ± 3/40 ± 9
AST (U/l)	99 ± 25/101 ± 27	79 ± 28/89 ± 11	89 ± 21/93 ± 24	97 ± 24/93 ± 19	100 ± 20/87 ± 29	103 ± 23/87 ± 27	86 ± 25/87 ± 29	94 ± 19/87 ± 27
GGT (U/l)	5.0 ± 0.0/5.0 ± 0.0	4.5 ± 1.6/5.0 ± 0.0	5.0 ± 0.0/5.0 ± 0.0	5.0 ± 0.0/5.0 ± 0.0	5 ± 0/5 ± 0	5 ± 0/5 ± 0	5 ± 0/5 ± 0	5 ± 0/5 ± 0
T-BIL (mg/dL)	0.2 ± 0.1/0.1 ± 0.0	0.1 ± 0.0/0.1 ± 0.0	0.2 ± 0.1/0.1 ± 0.0	0.1 ± 0.1/0.1 ± 0.0	0.2 ± 0.1/0.1 ± 0.1	0.2 ± 0.1/0.2 ± 0.1	0.2 ± 0.0/0.1 ± 0.1	0.2 ± 0.1/0.2 ± 0.1
T-PROT (g/dL)	5.8 ± 0.2/5.3 ± 0.2	5.2 ± 1.7/5.4 ± 0.2	5.6 ± 0.3/5.3 ± 0.2	5.6 ± 0.3/5.3 ± 0.2	5.7 ± 0.2/5.6 ± 0.2	5.9 ± 0.3/5.5 ± 0.1	5.7 ± 0.2/5.6 ± 0.2	5.7 ± 0.2/5.5 ± 0.1
GLU (mg/dL)	168 ± 16/177 ± 35	149 ± 48/179 ± 24	153 ± 22/156 ± 29	150 ± 20/163 ± 28	171 ± 21/189 ± 28	170 ± 28/181 ± 39	166 ± 26/189 ± 28	161 ± 25/181 ± 39
BUN (mg/dL)	21 ± 3/20 ± 2	20 ± 6/20 ± 2	19 ± 3/18 ± 2	21 ± 3/18 ± 4	22 ± 2/20 ± 3	20 ± 4/20 ± 2	22 ± 5/20 ± 3	20 ± 2/20 ± 2
CREA (mg/dL)	0.5 ± 0.0/0.4 ± 0.1	0.4 ± 0.1/0.3 ± 0.1	0.4 ± 0.1/0.4 ± 0.1	0.5 ± 0.1/0.4 ± 0.1	0.3 ± 0.0/0.4 ± 0.0	0.3 ± 0.0/0.4 ± 0.1	0.4 ± 0.1/0.4 ± 0.0	0.3 ± 0.0/0.4 ± 0.1
CHOL (mg/dL)	68 ± 10/64 ± 12	65 ± 22/60 ± 11	67 ± 12/56 ± 10	75 ± 12/66 ± 12	79 ± 12/59 ± 12	83 ± 15/63 ± 14	74 ± 13/59 ± 12	70 ± 10/63 ± 14
HDL (mg/dL)	52 ± 9/48 ± 7	51 ± 16/45 ± 9	52 ± 7/42 ± 8	55 ± 9/49 ± 7	61 ± 6/49 ± 10	63 ± 10/45 ± 8	58 ± 8/46 ± 8	55 ± 7/48 ± 11
TG (mg/dL)	66 ± 26/75 ± 26	65 ± 24/86 ± 22	60 ± 26/80 ± 37	64 ± 14/79 ± 19	73 ± 20/77 ± 23	72 ± 18/67 ± 22	62 ± 28/67 ± 14	66 ± 17/63 ± 14
P^3-^ (mg/dL)	8.3 ± 1.1/9.2 ± 0.6	7.5 ± 2.5/9.8 ± 0.6	8.0 ± 0.5/9.4 ± 0.8	8.7 ± 1.2/9.4 ± 0.6	8.1 ± 0.8/8.9 ± 0.6	8.0 ± 0.8/8.7 ± 0.4	7.7 ± 0.9/9.2 ± 0.9	7.9 ± 0.9/9.2 ± 0.4
Na^+^ (mEq/L)	141 ± 1/141 ± 1	127 ± 44/141 ± 0	140 ± 1/141 ± 1	139 ± 1/141 ± 1	140 ± 1/140 ± 1	140 ± 1/141 ± 1	140 ± 1/141 ± 1	140 ± 1/141 ± 1
K^+^ (mEq/L)	4.5 ± 0.2/5.2 ± 0.2	4.1 ± 1.4/5.0 ± 0.3	4.6 ± 0.4/5.0 ± 0.4	4.5 ± 0.2/4.9 ± 0.3	4.6 ± 0.3/5.0 ± 0.3	4.6 ± 0.2/5.0 ± 0.2	4.8 ± 0.6/5.2 ± 0.3	4.7 ± 0.5/4.9 ± 0.3
Cl^−^ (mEq/L)	104 ± 1.3/103 ± 2.5	93 ± 32.0/102 ± 2.1	105 ± 2.4/103 ± 1.4	103 ± 1.3/102 ± 0.9	104 ± 2/103 ± 1	103 ± 2/103 ± 1	103 ± 2/102 ± 2	103 ± 1/102 ± 2
Ca^2+^ (mg/dL)	10.5 ± 0.4/10.0 ± 0.3	9.6 ± 3.3/10.1 ± 0.2	10.4 ± 0.3/10.0 ± 0.2	10.1 ± 0.2*/10.1 ± 0.2	10.5 ± 0.2/10.8 ± 0.3	10.7 ± 0.2/10.6 ± 0.1	10.5 ± 0.2/10.6 ± 0.3	10.5 ± 0.3/10.5 ± 0.2
AMY (U/L)	1009 ± 159/1955 ± 261	893 ± 196/1996 ± 244	893 ± 196/1893 ± 254	936 ± 252/1884 ± 327	1038 ± 232/2078 ± 293	1195 ± 234/1970 ± 209	1012 ± 120/1963 ± 165	1100 ± 161/1895 ± 285
CPK (U/L)	689 ± 315/881 ± 283	593 ± 326/747 ± 291	593 ± 326/959 ± 418	807 ± 321/784 ± 209	805 ± 259/628 ± 362	831 ± 414/611 ± 390	661 ± 387/616 ± 303	753 ± 312/524 ± 254
LDH (U/L)	1527 ± 605/1878 ± 577	1336 ± 638/1466 ± 567	1336 ± 638/1771 ± 710	1672 ± 645/1775 ± 559	1595 ± 471/1223 ± 792	1639 ± 840/1140 ± 739	1275 ± 734/1338 ± 695	1488 ± 641/1079 ± 564

Data were presented as means ± standard deviation (n = 10, per group for male and female rats, after 28-day repeated oral administration). *P < 0.05 vs. the control group.

^a^ Control: equal volume of sterile water.

^b^ NRICM101 or 102, Low: 1.6 g/kg bw/day; Middle: 3.2 g/kg bw/day; High: 4.8 g/kg bw/day. Abbreviations: WBC, white blood cell; RBC, red blood cell; HGB, hemoglobin; HCT, hematocrit; MCV, mean corpuscular volume; MCH, mean corpuscular hemoglobin; MCHC, mean corpuscular hemoglobin concentration; PLT, platelet; PT, prothrombin time; APTT, activated partial thromboplastin time; INR, international normalized ratio; NEU, neutrophil; Lym, lymphocyte; MONO, monocyte; EOS, eosinophil; BASO, basophil; ALB, albumin; AST, aspartate aminotransferase; ALT, alanine aminotransferase; GGT, γ-glutamyl transferase; T-BIL, total bilirubin; T-PROT, total protein; GLU, glucose; BUN, blood urea nitrogen; CREA, creatinine; CHOL, cholesterol; HDL, high density lipoprotein; TG, triglyceride; P3-, inorganic phosphorus; Na^+^, sodium; K^+^, potassium; Cl^−^, chloride; Ca2+, calcium.

**TABLE 4:** Urine and urinary sediments analysis of NRICM101.

Index	Female								Male							
	Control ^a^		Low ^b^		Middle		High		Control		Low		Middle		High	
	Before	After	Before	After	Before	After	Before	After	Before	After	Before	After	Before	After	Before	After
Specific gravity	1.019 ± 0.003	1.018 ± 0.005	1.019 ± 0.002	1.017 ± 0.002	1.018 ± 0.003	1.017 ± 0.004	1.019 ± 0.005	1.018 ± 0.004	1.019 ± 0.005	1.019 ± 0.004	1.021 ± 0.006	1.019 ± 0.003	1.018 ± 0.003	1.020 ± 0.003	1.020 ± 0.003	1.019 ± 0.002
pH 7.0		1				1										
7.5	1	1		1		1			1							1
8.0	9	5	10	7	10	6	8	7	8	7	9	8	9	8	10	9
8.5		3		2		2	2	3	1	3	1	2	1	2		
Glucose																
-	10	10	10	10	10	10	10	10	10	10	10	10	10	10	10	10
Protein																
-	6	1	5	2	7	5	6	2	3		3	1	4		3	
±	4	5	5	6	3	3	2	4	4	1	3	1	3	2	4	
+		3		1		2	2	4	3	8	4	5	3	7	3	9
++		1		1						1		3				1
+++														1		
Bilirubin																
-	10	10	10	10	10	10	10	10	10	10	10	10	10	10	10	10
Urobilinogen																
-	10	9	10	10	10	9	9	9	10	10	9	9	10	9	9	10
+		1				1	1	1			1	1		1	1	
++																
+++																
Blood																
-	10	9	10	10	10	10	9	10	10	10	10	10	10	9	9	10
++		1					1							1	1	
Ketones																
-	6	6	5	9	5	9	5	8	5	4	4	5	7	5	3	3
±	3	4	5	1	5	1	2	2	3	5	2	1	3	4	6	5
+	1						3		2	1	4	4		1	1	2
Nitrite																
-	10	10	9	10	10	10	10	10	10	10	10	10	10	10	10	10
+			1													
Leukocyte																
-	5	3	4	5	5	6	6	7	3		4	1	3		7	
25	3	5	4	4	5	4	3	2	5	1	4		3		2	3
75	2	1	2	1			1	1	2	4	2	6	3	4	1	4
250		1								3		1	1	5		3
500										2		2		1		
Urinary sediments																
RBC(/HPF)^c^																
-	10	10	10	10	10	10	10	10	10	10	10	10	10	10	10	10
+																
WBC(/HPF)																
-	10	10	10	10	10	10	10	10	10	10	10	10	10	10	10	10
+																
Cast(/LPF)^d^																
-	10	10	10	10	10	10	10	10	10	10	10	10	10	10	10	10
+																
Cry.(/HPF)^e^																
-	1	1	1	1		1			2						1	
A									1							
C																
T	9	9	9	9	10	9	10	10	7		10		10		9	
Bac. (/LPF)																
-	9	9	9	10	9	10	8	10	5		7		9		9	
+	1	1	1		1		2		5		3		1		1	
SEC(/HPF)																
-	10	10	10	10	10	10	10	10	10	10	10	10	10	10	10	10
+																
NEC(/HPF)																
-	10	10	10	10	10	10	10	10	10	10	10	10	10	10	10	10
+																
Spermatozoa																
-	10	10	10	10	10	10	10	10	10	3	10	3	10	1	10	3
+										7		7		9		7
Urine output (ml, 6h)	1.5 ± 0.3	1.1 ± 0.4	1.3 ± 0.4	1.2 ± 0.6	1.6 ± 0.6	1.7 ± 0.9	1.4 ± 0.4	1.5 ± 0.6	1.5 ± 0.4	3.2 ± 1.2	2.2 ± 2.1	3.3 ± 1.6	1.8 ± 0.8	2.9 ± 1.1	1.5 ± 0.6	3.5 ± 1.5

^a^ Control: equal volume of sterile water.

^b^ NRICM101, Low: 1.6 g/kg bw/day; Middle: 3.2 g/kg bw/day; High: 4.8 g/kg bw/day.

^c^ HPF: high power field.

^d^ LPF: low power field.

^e^ Cry.: T: triple phosphate; A: amorphous phosphate; C: calcium oxalate; “-” negative, “±“: trace, “+“: slight, “++“: moderate, “+++“: severe; Data were presented as means ± standard deviation (n = 10, per group for male and female mouse or rats, 28-day after NRICM101 or 102 single dose administration). *P < 0.05 vs. the control group.

**TABLE 5:** Urine and urinary sediments analysis of NRICM102.

Index	Female								Male							
	Control ^a^		Low ^b^		Middle		High		Control		Low		Middle		High	
	Before	After	Before	After	Before	After	Before	After	Before	After	Before	After	Before	After	Before	After
Specific gravity	1.019 ± 0.002	1.016 ± 0.004	1.019 ± 0.003	1.017 ± 0.002	1.019 ± 0.003	1.015 ± 0.004	1.047 ± 0.089	1.018 ± 0.005	1.019 ± 0.003	1.021 ± 0.006	1.019 ± 0.003	1.035 ± 0.058	1.021 ± 0.004	1.017 ± 0.003	1.02 ± 0.005	1.02 ± 0.004
pH																
6.5		1				1										
7.0	1		1	2	1		1	1								
7.5			1	2			1	1		2						
8.0	8	4	8	5	9	6	6	7	7	4	6	2	7	5	9	8
8.5	1	5		1		3	2	1	3	4	4	8	3	5	1	2
Glucose																
-	10	10	10	10	10	10	10	10	10	10	10	10	10	10	10	10
±																
Protein																
-	3	1	2	1	7	3	4	4	3		1		1		1	
±	4	4	7	4	1	6	4	4	4		2		5		5	
+	3	5	1	5	2	1	2	2	3	3	7	2	4	5	3	5
++										7		7		3	1	4
+++												1		2		1
Bilirubin																
-	10	10	10	10	10	10	10	10	10	10	10	10	10	10	10	10
+																
Urobilinogen																
-	9	10	10	10	10	10	9	9	10	8	10	10	10	10	9	9
+	1									2						
++							1	1							1	1
+++																
Blood																
-	10	10	10	10	10	10	10	10	10	10	10	10	10	10	10	10
++																
Ketones																
-	5	7	7	5	8	9	9	6	8	1	6	3	7	2	5	2
±	5	3	2	4	2	1	1	3	2	5	4	4	3	5	4	3
+			1	1				1		4		3		3	1	5
Nitrite																
-	10	10	10	9	9	9	9	9	10	9	10	9	10	9	10	10
+				1	1	1	1	1		1		1		1		
++																
Leukocyte																
-	9	5	9	4	7	4	7	6	7		4		3		5	1
25	1	4	1	2	2	4	2	3	2	2	5		7	2	4	1
75		1		4	1	1	1	1	1	5	1	5		4	1	5
250						1				2		5		3		3
500										1				1		
Urinary sediments																
RBC(/HPF)^c^																
-	10	10	10	10	10	10	10	10	10	10	10	10	9	10	8	10
+													1		2	
WBC(/HPF)																
-	10	10	10	10	10	10	10	10	10	10	10	10	10	10	10	10
+																
Cast(/LPF)^d^																
-	10	10	10	10	10	10	10	10	10	10	10	10	10	10	10	10
+																
Cry.(/HPF)^e^																
-		1				4		2						1	1	
A																
C											1					
T	10	9	10	10	10	6	10	8	10	10	9	10	10	10	9	10
Bac. (/LPF)																
-	10	10	8	10	10	10	9	10	9	10	9	10	8	10	8	10
+			2				1				1		1		1	
++									1				1		1	
SEC(/HPF)																
-	10	10	10	10	10	10	10	10	10	10	10	10	10	10	10	10
+																
NEC(/HPF)																
-	10	10	10	10	10	10	10	10	10	10	10	10	10	10	10	10
+																
Spermatozoa																
-	10	10	10	10	10	10	10	10	10	1	10	2	10	1	10	2
+										9		8		9		8
Urine output (ml, 6h)	1.7 ± 0.8	1.4 ± 1.0	1.9 ± 1.2	1.5 ± 0.7	1.7 ± 0.6	1.7 ± 0.7	1.5 ± 0.4	1.8 ± 1.0	1.4 ± 0.6	2.9 ± 1.5	1.5 ± 0.7	2.4 ± 0.9	1.5 ± 0.8	2.5 ± 1.1	1.6 ± 0.9	2.6 ± 1.4

^a^ Control: ^a^qual volume of sterile water.

^b^ NRICM, 102^b^ Low: 1.6 g/kg bw/day; Middle: 3.2 g/kg bw/day; High: 4.8 g/kg bw/day.

^c^ HPF: high^c^power field.

^d^ LPF: low ^d^ower field.

e Cry.: T: triple phosphate; A: amorphous phosphate; C: calcium oxalate; “-” negative, “±“: trace, “+“: slight, “++“: moderate, “+++“: severe; Data were presented as means ± standard deviation (n = 10, per group for male and female mouse or rats, 28-day after NRICM101 or 102 single dose administration). *P < 0.05 vs. the control group.

#### 3.2.4 Histopathological analysis of the vital organs

**FIGURE 6:**
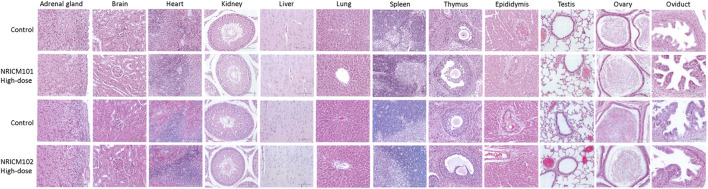
Histopathology representative photomicrographs of adrenal gland, brain, heart, kidney, liver, lung, spleen, thymus, epididymis, testis, ovary, and oviduct sections of mice treated orally with NRICM101 or 102 (4.8 g/kg bw/day) for 28 days (×400 magnification).

Observations from necropsies performed on rats administered NRICM101 and 102 showed no discernible pathological abnormalities in vital organs across all groups after 28 days of repeated administration (Figure 6).

### 3.3 Genotoxicity study

#### 3.3.1 Ames test

**FIGURE 7:**
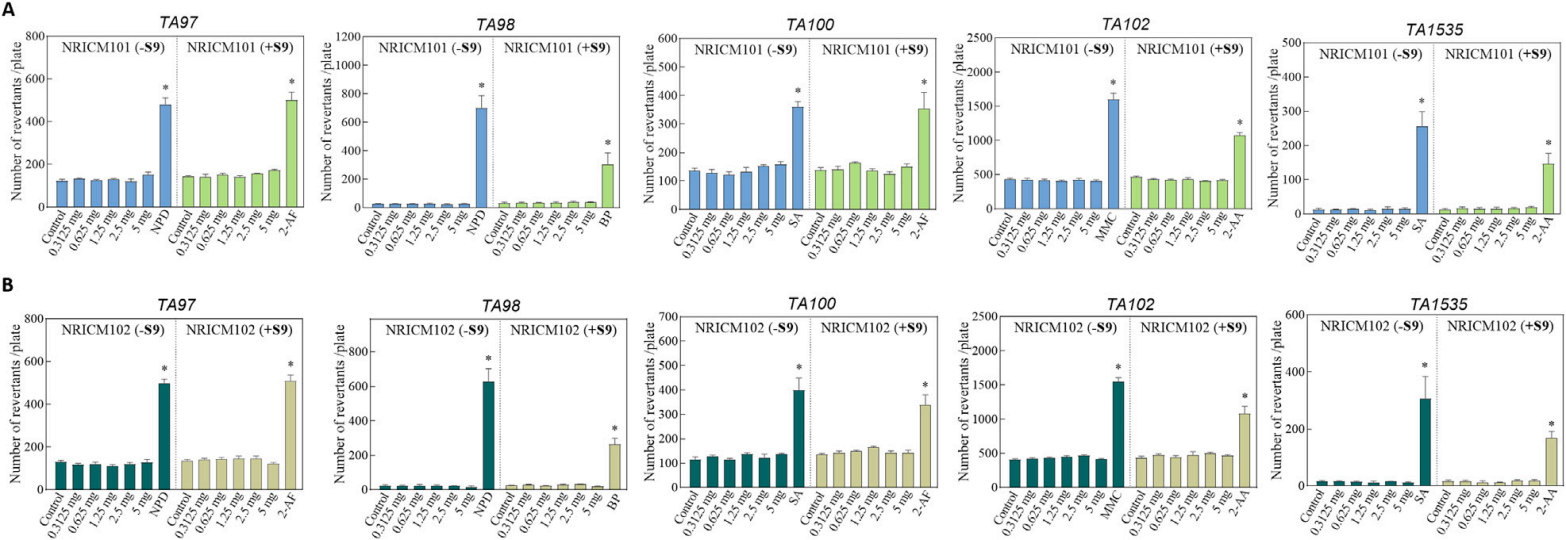
**(A)** Effect of NRICM101 on mutagenicity using the Ames test. The mutagenic potential of NRICM101 was evaluated in *S. typhimurium* strains TA97a, TA98, TA100, TA102, and TA1535 in the presence or absence of S9 metabolic activation. Each test mixture included 100 µL of NRICM101 or NRICM102 and 0.1 mL of bacterial suspension added to molten top agar containing 0.5 mM histidine/biotin. After 48 h incubation, revertant colonies were counted. For S9-treated groups (+S9), 0.5 mL of S9 mix was included; sodium phosphate buffer was used in groups without S9 (-S9). Sterile water served as the negative control. Positive controls for -S9 groups: 10.0 μg/plate of 4-nitro-o-phenylenediamine (NPD) for TA97a and TA98; 0.4 μg/plate of sodium azide (SA) for TA100 and TA1535; 0.5 μg/plate of mitomycin C (MMC) for TA102; Positive controls for + S9 groups: 4.0 μg/plate of 2-aminofluorene (2-AF) for TA97a and TA100; 4.0 μg/plate of benzo[α]pyrene (BP) for TA98; 4.0 μg/plate of 2-aminoanthracene (2-AA) for TA102 and TA1535. Data are presented as mean ± standard deviation (n = 3). *P < 0.05 vs. control group. **(B)** Effect of NRICM102 on mutagenicity using the Ames test. The mutagenic potential of NRICM102 was evaluated in *S. typhimurium* strains TA97a, TA98, TA100, TA102, and TA1535 in the presence or absence of S9 metabolic activation. For S9-treated groups (+S9), 0.5 mL of S9 mix was included; sodium phosphate buffer was used in groups without S9 (-S9). Sterile water served as the negative control. Positive controls for -S9 groups: 4-nitro-o-phenylenediamine (NPD) for TA97a and TA98; sodium azide (SA) for TA100 and TA1535; mitomycin C (MMC) for TA102; Positive controls for + S9 groups: 2-aminofluorene (2-AF) for TA97a and TA100; benzo[α]pyrene (BP) for TA98; 2-aminoanthracene (2-AA) for TA102 and TA1535. After 48 h incubation, revertant colonies were counted. Data are presented as mean ± standard deviation (n = 3). *P < 0.05 vs. control group.

In the mutagenicity potential test, *S. Typhimurium* strains (TA97a, TA98, TA100, TA102, and TA1535) were treated with NRICM101 or 102 (0.3125, 0.625, 1.25, or 2.5 mg/plate) with or without metabolic activator S9 mix. Mutagenicity was determined by comparing the number of revertant colonies to the negative control. Positive mutagenicity was indicated when the treatment increased the revertant colonies by more than two-folds over those observed with negative control. As shown in Figure 7 (Supplementary Table S6), the positive controls consistently showed significantly higher revertant colonies than the negative control, meeting the criteria for positive mutagenicity. However, at all doses of NRICM101 and 102, the revertant colonies did not reach a minimum of a two-fold multiple of the negative control, with or without S9 activation.

#### 3.3.2 MLA results

**FIGURE 8:**
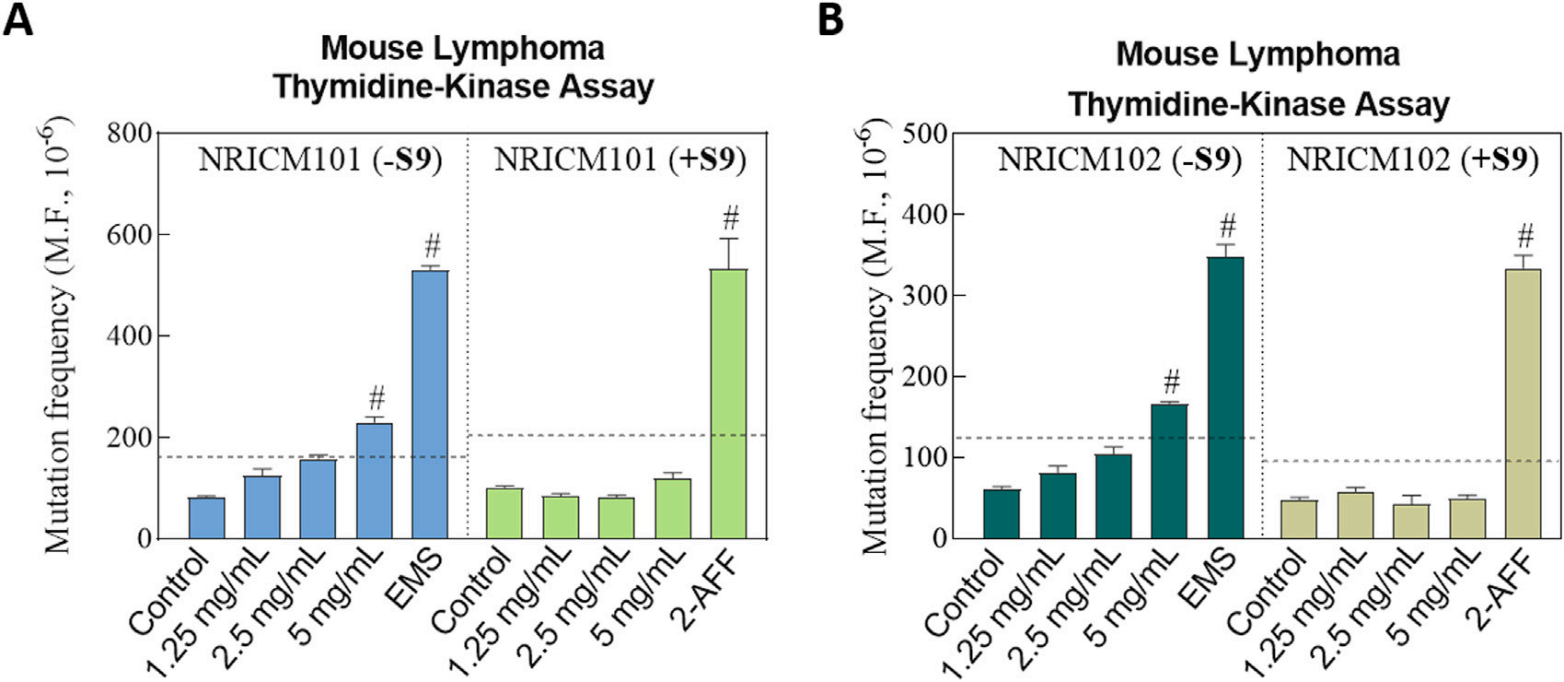
Effect of NRICM101 or 102 on toxicity and mutagenicity using the mouse lymphoma assay (MLA) with and without S9 metabolic activation.L5178Y TK^+/−^ cells were treated with NRICM101 **(A)** or NRICM102 **(B)** at 1.25, 2.5, and 5.0 mg/mL. Treatments were conducted in the presence or absence of S9 mix. Cells were incubated for 12 days to allow for colony formation. D-PBS was used as the negative control, while ethyl methanesulfonate (EMS, 0.32 mg/mL) and N-(2-fluorenyl)acetamide (2-AAF, 0.2 mg/mL) served as positive controls for the with S9 (+S9) or without S9 (-S9) conditions, respectively. After treatment, cells were plated at a density of two cells/well in non-selective medium or 2000 cells/well in trifluorothymidine (TFT)-containing selective medium. Colony formation was scored manually according to OECD Guideline No. 490, with mutant colonies categorized as small or large. Mutation frequency (MF) was calculated by the following equation. MF = cloning efficiency (C.E.%)/Plating efficiency (P.E.%). C.E.% = [-ln(P0)/2000]×100 (selective medium). P.E.% = [-ln(P0)/2]×100 (non-selective medium). P(0) = number of empty wells/total wells plated. A ≥2-fold increase in MF relative to the negative control was considered a mutagenic response, indicating as *#*. Data are presented as mean ± standard deviation (n = 3).

The potential toxicity and mutagenicity of NRICM101 and 102 were assessed in L5178Y/TK^+/−^ mouse lymphoma cells with and without the metabolic activator S9 mix. Preliminary cytotoxicity tests indicated no significant cytotoxic effects of NRICM101 or 102 at doses of 1.25, 2.5, or 5.0 mg/mL in L5178Y/TK^+/−^ mouse lymphoma cells (Figure 8; Supplementary Table S7). In the negative control group, P.E.%, C.E.%, and MF were all within acceptable ranges according to the OECD test guidelines. Positive mutagenicity was indicated when the treatment increased MF by more than two-fold over that of the negative control. As positive controls, EMS (0.32 mg/mL) and 2-AAF (0.2 mg/mL), corresponding to the absence and presence of S9, respectively, both showed more than a two-fold increase compared to the negative control groups in the presence and absence of S9, validating the assay. In the presence of the S9 mix, all three doses of NRICM101 or 102 (1.25, 2.5, and 5.0 mg/mL) displayed acceptable MF values (NRICM101: 84.1 ± 4.6, 81.0 ± 5.1, and 119.2 ± 11.6; NRICM102: 58.3 ± 4.2, 42.0 ± 11.1, and 48.9 ± 4.8) when compared to the negative control (NRICM101: 101.2 ± 2.8; NRICM102: 48.5 ± 3.1). In the absence of the metabolic activator S9 mix, the two lower doses of NRICM101 or 102 (1.25 and 2.5 mg/mL) also yielded acceptable MF values (NRICM101: 124.9 ± 13.3 and 157.8 ± 7.4; NRICM102: 81.3 ± 8.0 and 104.0 ± 8.6) when compared to the negative control (NRICM101: 81.6 ± 2.7; NRICM102: 61.6 ± 2.4). However, at the 5.0 mg/mL concentration, both NRICM101 and 102 exhibited MF values of 229.9 ± 10.8 and 166.7 ± 2.4 mutants, respectively, more than two-fold of that recorded with the negative control group.

#### 3.3.3 Micronuclei assay in the mouse peripheral blood

**FIGURE 9:**
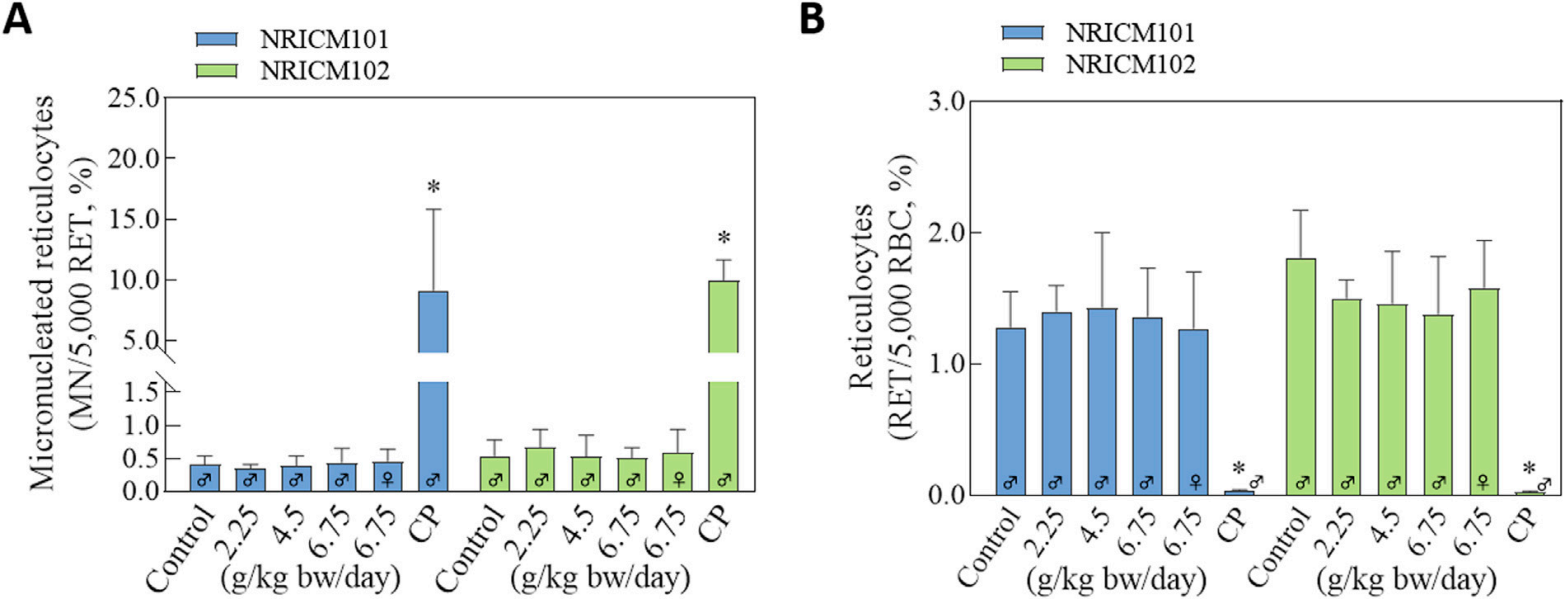
Effects of NRICM101 or 102 on the percentages of reticulocytes and the frequencies of micronucleated reticulocytes in ICR mice. ICR mice were administered sterile water (negative control), cyclophosphamide (100 mg/kg bw; positive control), or NRICM101/NRICM102 at low (2.25 g/kg bw/day), middle (4.50 g/kg bw/day), or high (6.75 g/kg bw/day) doses. At 48 h post-administration, peripheral blood was collected for micronucleus analysis. Micronucleated reticulocytes (MN-RET) **(A)** and Reticulocytes (RET) **(B)** were quantified by flow cytometry following staining with FITC-conjugated anti-CD71 and propidium iodide (PI). Data are presented as mean ± standard deviation (n = 5). *P < 0.05 vs. negative control group.

NRICM101 and 102 were further investigated using the *in vivo* erythrocyte micronucleus test in ICR mice to assess potential chromosomal damage (Figure 9; Supplementary Table S8). The positive control group that was administered cyclophosphamide showed a decreased ratio of reticulocytes to total erythrocytes and an increased frequency of micronucleated polychromatic erythrocytes. After NRICM102 administration, the average ratios of micronucleated reticulocytes and reticulocytes in the negative control group were 0.54% ± 0.24% and 1.81% ± 0.36%, respectively. At the dosages of 2.25, 4.5, and 6.75 mg/kg bw of NRICM102, the ratios of micronucleated reticulocytes were 0.67% ± 0.27%, 0.53% ± 0.32%, 0.51% ± 0.15% (male), and 0.59% ± 0.35% (female), respectively, with corresponding reticulocyte ratios of 1.5% ± 0.14%, 1.46% ± 0.40%, 1.38% ± 0.36% (male), and 1.58% ± 0.44% (female), respectively. Similarly, after NRICM101 administration, the average ratios of micronucleated reticulocytes and reticulocytes in the negative control group were 0.41% ± 0.13% and 1.28% ± 0.27%, respectively. At the dosages of 2.25, 4.5, and 6.75 mg/kg bw of NRICM101, the ratios of micronucleated reticulocytes were 0.35% ± 0.06%, 0.39% ± 0.15%, 0.44% ± 0.21% (male), and 0.46% ± 0.18% (female), respectively, with corresponding reticulocyte ratios of 1.4% ± 0.2%, 1.43% ± 0.57%, 1.36% ± 0.37% (male), and 1.27% ± 0.43% (female), respectively. Across all doses, no significant differences were observed in micronucleated reticulocyte or reticulocyte ratios compared to controls.

## 4 Discussion

NRICM101 and 102 are supplements used as a therapeutic measure against COVID-19; therefore, understanding their toxicity is essential to ensure their safety. In the toxicological evaluation study, we comprehensively evaluated the potential toxicity and genotoxicity of NRICM101 and 102 *in vitro* and *in vivo*. In the acute oral toxicity study, no significant differences were observed in either sex when administered 5 g/kg bw of NRICM101 or 102, and no mortality-related effects or clinical signs were attributed to the test materials. Furthermore, no discernible variations were observed in body weight between these groups at any time point during the acute oral toxicity study. Blood serum biochemistry analysis, specifically ALT, AST, BUN, and CREA levels, revealed that NRICM101 and 102 had similar effects on basic liver and renal function. Male SD rats treated with NRICM101/102 exhibited no significant differences in AST, ALT, BUN, or CREA levels when compared to the control group. The only parameter affected by NRICM101 and 102 were the AST levels, which were lower than those of the control group. Additionally, based on the results of the visual organ inspection examination, no clinical abnormalities associated with the administration of NRICM101 and 102 were observed. As a result, it can be concluded that NRICM101 and 102 are safe for acute oral administration at a single dose of 5 g/kg bw, both in ICR mice and SD rats.

We also verified the repeated dose toxicity of NRICM101 and 102 by conducting a 28-day subchronic toxicity test in rats. During the experiment, daily repeated oral administration with NRICM101 or 102 for 28 days showed no evident abnormal clinical signs, and deaths were not recorded at any dose or in the control group. The impact of NRICM101 and NRICM102 on body weight and percent body weight change was also examined in both males and females. While significant differences were observed in some groups for both NRICM101 and 102, notably, these fluctuations were rapidly normalized, with animals regaining weight at rates similar to the control group in subsequent weeks. Given the sporadic nature of these changes and their absence of a dose-dependent trend, these changes may not be associated with the test materials. Similarly, notable reductions were observed in food consumption in certain groups. However, these changes were sporadic and lacked a dose-dependent pattern, indicating that the reductions were not primarily attributed to the test substances. Therefore, overall, these results suggest that NRICM101 and NRICM102 had minimal and temporary effects on body weight, body weight change, and food consumption, with no clear evidence of adverse impacts. Although ROW analysis showed significant differences in the adrenal gland in female rats that were administered a middle dose of NRICM102 and male rats that were administered a low dose of NRICM102 compared to the control group, these results may be attributed to the normal physiological changes in rats or not treatment-related effects.

Moreover, hematological analysis revealed no severe effects of NRICM101 and NRICM102 in male and female rats (Table 5). However, some parameters were still affected by either NRICM101 and/or 102. In the case of low-dose NRICM101, no significant changes were observed in hematological indices in both male and female rats. This suggests that at this dose, NRICM101 had minimal influence on these parameters in either gender. Upon administering a middle dose of NRICM101 to female rats, significant changes were noted, including increased RBC counts (7.32 ± 0.36 × 10^6^/μL) and HCT levels (42.9% ± 2.1%), along with a decrease in MCHC (33.9 ± 0.9) levels compared to the control group (which had RBC counts of 6.77 ± 0.40 × 10^6^/μL, HCT levels of 39.8% ± 2.3%, and MCHC levels of 34.9 ± 0.8 g/dL). This suggests that middle-dose NRICM101 can affect specific hematological parameters in female rats but does not significantly impact others. In contrast, middle-dose NRICM101 had no significant effects on hematological parameters in male rats, indicating gender-related differences in response. Upon administration of a high dose of NRICM101 in female rats, significant increases were observed in RBC counts (7.32 ± 0.44 × 10^6^/μL), HCT levels (43.8% ± 2.5%), and HGB levels (15.0 ± 0.6 g/dL); however, a decrease in calcium (Ca^2+^) levels (10.1 ± 0.2 mg/dL) was also observed, compared to the control group (RBC: 6.77 ± 0.40 × 10^6^/μL, HCT: 39.8% ± 2.3%, HGB: 13.9 ± 0.7 g/dL, Ca^2+^: 10.5 ± 0.4 mg/dL). This suggests that high-dose NRICM101 can significantly impact these hematological parameters in female rats, with the decrease in calcium levels being particularly noteworthy. In contrast, high-dose NRICM101 in male rats led to significant increases in PT (13.4 ± 2.2 s) and INR (1.2 ± 0.2) compared to the control group (PT: 10.7 ± 0.4 s, INR: 1.2 ± 0.2), without significant changes in other hematological parameters. These results indicate that high-dose NRICM101 might affect blood coagulation parameters in male rats.

Shifting to NRICM102, only low-dose-treated male rats exhibited a significant decrease in PLT levels (1104 ± 132 × 10^3^/μL) compared to the control group (PLT: 1274 ± 155 × 10^3^/μL), with no significant changes in other hematological parameters across all NRICM102-administered groups. This suggests that low-dose NRICM102 specifically impacted platelet levels in male rats whereas other hematological parameters remained relatively unaffected. In summary, the hematological effects of NRICM101 and 102 vary depending on the dose, gender, and specific hematological parameters considered. These findings provide insights into the potential impacts of the NRICM101 or 102 on blood-related parameters in rats.

Necropsies conducted on rats subjected to NRICM101 and 102 revealed a notable absence of pathological abnormalities in vital organs, irrespective of the administered doses, following a 28-day repeated administration period. Additionally, in-depth necropsy analysis indicated no dose-dependent gross morphological differences of toxicological significance. Microscopic examination of the examined organs in both control and high-dose treatment groups showed no noteworthy histopathological alterations (Figure 6). Hence, the aforementioned evidence provide important insights into the safety profile of NRICM101 and 102, suggesting that these substances did not cause adverse pathological effects on vital organs or major histopathological changes in the examined organs. However, it is acknowledged that some physiological variations occurred, which were considered within the normal range for rats or unrelated to the treatment.

The recommended human therapeutic dose of NRICM101 and NRICM102 is 300 mL/day of decoction (15 g/day lyophilized powder), approximately 0.25 g/kg/day for a 60 kg adult. Using allometric scaling (conversion factor 0.625 for rats), this translates to 1.6 g/kg/day in rats. Accordingly, our 28-day subchronic oral toxicity study used 1.6 g/kg/day as the reference dose, with additional groups receiving 2× (3.1 g/kg/day) and 3× (4.8 g/kg/day), showing no treatment-related deaths or histopathological changes. Furthermore, a single oral dose of 5 g/kg in both SD rats and ICR mice showed no mortality or observable signs of toxicity. Importantly, the *in vivo* micronucleus assay, which assesses systemic genotoxicity under physiologically relevant conditions, showed no genotoxic effects even at doses up to 6.75 g/kg/day. Together, the *in vivo* studies provide definitive evidence under clinically relevant conditions and demonstrate a wide safety margin between the therapeutic dose and the highest tested exposures.

Genotoxicity refers to the ability of substances to directly or indirectly damage DNA, potentially leading to mutations without necessarily causing cytotoxicity. In this study, we evaluated the genotoxic potential of NRICM101 and NRICM102 using three established assays, including the bacterial reverse mutation assay (Ames test), the mouse lymphoma assay (MLA), and the *in vivo* micronucleus test, which are widely accepted in toxicological evaluations (Hayashi, 2022). The Ames test showed that with and without S9, NRICM101 or 102 did not increase the values of revertants over spontaneous revertants in 5 *S. typhimurium* strains, TA97a, TA98, TA100TA102, and TA1535 at all test doses. Therefore, the Ames test of NRICM101 and 102 were negative, and these substances were concluded to possess a low probability of inducing mutations. In the MLA assay, both formulations tested positive only at the highest concentration (5.0 mg/mL) without S9 activation, but were negative with S9, suggesting potential direct-acting genotoxicity in the absence of metabolic activation. This implies that the observed genotoxicity may stem from direct-acting compounds or non-specific stress responses, such as oxidative stress or cytotoxicity, at high concentrations. The negative results with S9 further suggest possible detoxification by metabolic enzymes or the involvement of secondary mechanisms rather than true genotoxic effects. These results may also imply potential detoxification by metabolic enzymes. However, further investigation is needed to clarify the underlying mechanisms. In contrast, the *in vivo* micronucleus assay conducted in ICR mice showed no significant increase in micronucleated reticulocytes, reflecting a lack of systemic clastogenic or aneugenic effects at therapeutically relevant doses. Our results showed the discrepancy between the negative Ames and micronucleus tests and the positive MLA result without S9. It may be due to the longer duration of exposure to the substances in the MLA assay (12 days) compared to the shorter duration of the Ames test (2 days). The duration of the assay has been suggested as an important factor for their genotoxicity evaluation (Pinter et al., 2020). Additionally, the difference in cell types, bacteria used in the Ames test and mammalian cells used in MLA assay, may also contribute to these varying results. The MLA uses mammalian cells (L5178Y TK^+/−^ mouse lymphoma cells), which are more sensitive to a broader range of genotoxic mechanisms than bacterial cells used in the Ames test. However, the micronucleus assay, performed *in vivo* in mammalian erythrocytes, detects clastogenic (chromosomal breakage) and aneugenic (chromosome loss) events, suggesting no significant effect on systemic genotoxicity evaluation under physiologically relevant conditions. The results of our study demonstrated no signs of cytotoxicity or histopathological changes. Additionally, both the Ames test and the *in vivo* micronucleus assay yielded negative results for genotoxicity. However, we did observe high-dose MLA positivity in the absence of S9. According to OECD guidelines, *in vitro* genotoxicity findings observed only at high concentrations without metabolic activation should be interpreted with caution and considered in the context of *in vivo* results and overall toxicological profiles. Nevertheless, additional *in vivo* genotoxicity evaluations and pharmacokinetic studies are needed to further investigate the safety of NRICM101 and NRICM102 under conditions of prolonged or high-dose use.

NRICM101 and NRICM102 both incorporates a blend of 10 botanical plants, sharing five common and five distinct. They both enrich in flavonoids, particularly baicalin, wogonoside, and quercetin 3-rhamnoside (Tsai et al., 2021; Wei et al., 2022), which have been reported to exert beneficial hematological effects, including anti-leukemic activity, antioxidant protection, and immune modulation but there are poorly reported for their genotoxicity. Baicalin, a predominant metabolite in both NRICM101 and 102 which may mainly from *S. baicalensis*, has been extensively studied for its anti-inflammatory, antiviral (including anti-SARS-CoV-2), antioxidant, and anticancer properties (Dinda et al., 2023; Huynh et al., 2025). These effects are partly attributed to its capacity to scavenge reactive oxygen species (ROS) and modulate signaling pathways such as NF-κB, JAK/STAT, and MAPK (Hu et al., 2022). While baicalin demonstrates pro-apoptotic activity against various cancer cell lines, including hematologic malignancies, it spares normal leukocytes, suggesting selective toxicity (Bernasinska-Slomczewska et al., 2024). However, direct evidence regarding its genotoxicity is limited. Wogonoside exhibits anti-inflammatory and anticancer effects through inhibition of NF-κB and ATF2 signaling pathways (Xu et al., 2021). It has also been shown to induce cell cycle arrest in acute myeloid leukemia cells (Narayanankutty et al., 2021). Nevertheless, little is known about its potential genotoxicity. Quercitrin, a glycosylated form of quercetin, has been reported to exhibit antioxidant, anti-inflammatory, and antiviral activities (Ho et al., 2024). Although quercetin aglycone has been associated with genotoxic effects in some *in vitro* systems, quercitrin and other glycosides generally show no genotoxicity *in vivo* and may even exhibit genoprotective effects through DNA repair enhancement and ROS scavenging (O'Connor et al., 2009). Together, to the best of our knowledge, the primary metabolites in NRICM101 and NRICM102 that could potentially pose a genotoxic risk at physiologically relevant concentrations remain largely unidentified, and further investigation is warranted.

## 5 Conclusion

With this study, we primarily aimed to evaluate the safety and genotoxicity of NRICM101 and 102. First, in the acute oral toxicity study, where a single dose of 5 g/kg bw of NRICM101 or 102 was administered to both ICR mice and SD rats, no significant differences were observed between the sexes. No mortality-related effects or clinical signs attributed to the substances were observed, and body weight remained consistent. Blood serum biochemistry analysis revealed similar effects on liver and renal function parameters, with a notable decrease in AST levels in male rats. Second, the 28-day repeated subchronic toxicity study in rats indicated that daily repeated oral administration of NRICM101 or 102 did not lead to observable abnormal clinical signs, deaths, or significant variations in body weight. Some differences in ROWs were noted but were attributed to normal physiological changes or unrelated factors. Hematological analysis revealed dose and gender-dependent effects, impacting specific parameters, implying potential effects on blood parameters, though generally within acceptable limits. Third, genotoxicity was assessed using the Ames test, the mouse lymphoma assay (MLA), and the *in vivo* micronucleus test. NRICM101 and NRICM102 did not induce acute or subchronic toxicity in rats at clinically relevant doses and were negative for mutagenicity in the Ames test and for clastogenicity in the *in vivo* micronucleus assay. However, both formulations exhibited positive responses in the mouse lymphoma assay at high concentrations without metabolic activation, indicating a potential for direct genotoxic effects under certain *in vitro* conditions. These observations emphasize the importance of metabolic context and concentration relevance in genotoxicity evaluations. Further long-term *in vivo* studies and human pharmacokinetic assessments are needed to thoroughly characterize the genotoxic potential, particularly in scenarios involving prolonged and high-dose administration.

## Data Availability

The original contributions presented in the study are included in the article/Supplementary Material, further inquiries can be directed to the corresponding authors.
