# Supplementary material for: Toxicological assessment of novel Anti-COVID traditional Chinese medicine formulae NRICM101 and NRICM102: a comprehensive study on safety and genotoxicity
Source: Front Pharmacol. 2025 Sep 17;16:1596369. doi: 10.3389/fphar.2025.1596369 (PMC12484172; doi:10.3389/fphar.2025.1596369)
Supplement: Supplementary file 1 [file DataSheet1.pdf]

Supplementary information

Toxicological Assessment of Novel Anti-COVID Traditional Chinese Medicine Formulae NRICM101 and NRICM102: A Comprehensive Study on Safety and Genotoxicity

Authors:

Chun-Tang Chiou[1,†], Chao-Lin Chang[2,†], Yu-Hwei Tseng[1,3], Geng-You Liao[4], Jiunn-Wang Liao[5], Yuh-Chiang Shen[1], Wen-Chi Wei[1], Keng-Chang Tsai[1,6], Yu-Ching Huang[1], Wen-Chiung Chang[1], Wen-Fei Chiou[1], Chia-Ching Liaw[1,7,8,9,10*], and Yi-Chang Su[1*]

[1]National Research Institute of Chinese Medicine, Ministry of Health and Welfare; Taipei 112, Taiwan

[2]Food Industry Research and Development Institute, Hsinchu 300, Taiwan

[3]Department of Public Health, National Cheng Kung University, Tainan 704, Taiwan

[4]Institute of Physiology, School of Medicine, National Yang Ming Chiao Tung University, Taipei 112304, Taiwan

[5]Graduate Institute of Veterinary Pathobiology, National Chung Hsing University, Taichung 402, Taiwan

[6]Ph.D. Program in Medical Biotechnology, College of Medical Science and Technology, Taipei Medical University, Taipei 1103, Taiwan

[7]Department of Biochemical Science and Technology, National Chiayi University, Chiayi 600, Taiwan

[8]Graduate Institute of Natural Products, Kaohsiung Medical University, Kaohsiung 807, Taiwan

[9]Department of Pharmacy, School of Pharmaceutical Sciences, National Yang Ming Chiao Tung University, Taipei 112, Taiwan

School of Chinese Medicine, College of Medicine, National Yang Ming Chiao Tung University, Taipei 112, Taiwan

[†]These authors contributed equally to this work as co-first authors.

***Corresponding Authors:**

Dr. Chia-Ching Liaw; Division of Chinese Materia Medica Development, National Research Institute of Chinese Medicine, Ministry of Health and Welfare, No. 155-1 Li-Nong Street, Section 2, Taipei City112, Taiwan. Tel.: +886-2-28201999, ext.2621; Email: liawcc@nricm.edu.tw; Dr. Yi-Chang Su, National Research Institute of Chinese Medicine, Ministry of Health and Welfare, No. 155-1 Li-Nong Street, Section 2, Taipei 112, Taiwan. Tel.: +886-2-28201999, ext. 3101; Email: sychang@nricm.edu.tw

Content

Table S1. Effect of NRICM101 or 102 on body weight gain in the single doe acute oral toxicity test.
Table S2. Effect of NRICM101 or 102 on serum biochemical indexes in rats in the single-dose acute oral toxicity test.
Table S3. Effect of NRICM101 or 102 on SD rat body weight in the repeated subacute oral toxicity test.
Table S4. Effect of NRICM101 or 102 on body weight change in the repeated subacute oral toxicity test.
Table S5. Effect of NRICM101 or 102 on food consumption in the repeated subacute oral toxicity test.
Table S6. The mutagenicity of NRICM101 or 102 in *S. typhimurium* strains in the absence or presence of S9 mix.
Table S7. The toxicity and mutagenicity of NRICM101 or 102 in L5178YTk+/- cells with/without S9 fraction by the mouse lymphoma thymidine-kinase assay (MLA).
Table S8. Effects of NRICM101 or 102 on the percentages of reticulocytes and the frequencies of micronucleated reticulocytes in ICR mice.

Table S1. Effect of NRICM101 or 102 on body weight gain in the single doe acute oral toxicity test.

Treatment	Body weight (g) ICR mouse					
	Day 1	Day 7	Day 14	Day 1	Day 7	Day 14
		Female			Male	
Control[a]	25.58 ± 1.0	28.28 ± 1.0	29.42 ± 1.0	32.08 ± 1.7	35.92 ± 1.5	37.54 ± 1.6
NRICM101[b]	25.90 ± 0.8	28.90 ± 2.6	30.42 ± 2.7	33.10 ± 1.1	36.38 ± 1.5	37.70 ± 1.9
		Female			Male	
Control	26.50 ± 2.2	29.50 ± 1.8	31.40 ± 2.3	31.60 ± 1.4	34.80 ± 1.8	36.30 ± 1.8
NRICM102[b]	26.02 ± 1.2	28.96 ± 2.4	30.86 ± 2.4	32.68 ± 2.1	36.82 ± 2.4	39.04 ± 2.4
**Treatment**	**Body weight (g) SD rats**					
	**Day 1**	**Day 7**	**Day 14**	**Day 1**	**Day 7**	**Day 14**
		Female			Male	
Control	220.46 ± 7.9	240.12 ± 15.4	256.72 ± 17.7	278.24 ± 5.8	328.94 ± 10.4	367.46 ± 17.3
NRICM101	220.10 ± 6.3	238.54 ± 11.1	252.64 ± 10.7	282.28 ± 5.8	333.9 ± 9.6	376.34 ± 9.7
NRICM102	223.12 ± 7.2	246.9 ± 11.5	257.90 ± 14.7	280.86 ± 5.9	337.68 ± 9.5	391.12 ± 12.6

Data were presented as means ± SD (n = 5, per group for male and female ICR mouse or rats, after 14-day administration). *$P < 0.05$ vs. control group.; [a] Control: equal volume of sterile water; [b] NRICM101 and 102: 5 g/kg bw.

Table S2. Effect of NRICM101 or 102 on serum biochemical indexes in rats in the single-dose acute oral toxicity test.

Treatment	Serum biochemical indexes in SD rats							
	AST	ALT	BUN	CREA	AST	ALT	BUN	CREA
	Female				Male			
Control[a]	87.8 ± 9.2	34.0 ± 9.4	22.4 ± 5.2	0.5 ± 0.1	90.8 ± 26.6	37.6 ± 8.7	21.8 ± 6.4	0.5 ± 0.1
NRICM101[b]	73.0 ± 9.6*	33.2 ± 3.0	17.2 ± 1.5	0.4 ± 0.1	72.6 ± 5.5	32.8 ± 3.3	20.4 ± 3.6	0.5 ± 0.0
NRICM102	63.0 ± 6.0*	29.6 ± 4.5	20.4 ± 2.5	0.4 ± 0.1	87.2 ± 7.6	36.4 ± 4.2	16.8 ± 1.9	0.5 ± 0.0

Data were presented as means ± SD (n = 5, per group for male and female mouse or rats, 14-day after NRICM101 or 102 single dose administration). *$P < 0.05$ vs. the control group. Abbreviations: AST, aspartate aminotransferase; ALT, alanine aminotransferase; BUN, blood urea nitrogen, CREA, creatinine. [a] Control: equal volume of sterile water; [b] NRICM101 and 102: 5 g/kg bw.

Table S3. Effect of NRICM101 or 102 on SD rat body weight in the repeated subacute oral toxicity test.

Treatment	Body weight (g) SD rats									
	NRICM101					NRICM102				
Week	0	1	2	3	4	0	1	2	3	4
	Female					Female				
Control [a]	152.5 ± 9.5	191.6 ± 12.5	213.0 ± 16.6	234.3 ± 17.4	245.2 ± 18.3	152.9 ± 6.4	179.2 ± 7.5	207.0 ± 8.7	227.4 ±9.2	236.5 ± 7.7
Low [b]	151.1 ± 10.3	194.2 ± 11.2	215.2 ± 15.0	229.0 ± 20.4	244.2 ± 21.6	152.3 ± 7.9	176.6 ± 8.5	200.7 ± 12.3	219.4 ± 12.8	231.4 ± 13.0
Middle	151.5 ± 9.4	187.8 ± 11.3	208.4 ± 14.0	223.6 ± 16.8	236.5 ± 19.6	152.0 ± 8.9	180.1 ± 12.4	201.8 ± 15.6	219.6 ± 14.8	233.1 ± 17.0
High	151.1 ± 11.6	189.5 ± 17.4	211.1 ± 24.7	229.6 ± 34.2	240.0 ± 34.7	153.7 ± 8.6	184.4 ± 10.9	210.5 ± 15.4	227.5 ± 13.5	240.2 ± 17.6
	Male					Male				
Control	172.3 ± 9.7	256.7 ± 15.9	317.6 ± 20.4	369.2 ± 27.9	404.6 ± 33.7	170.7 ± 13.3	227.6 ± 13.7	294.2 ± 15.7	350.6 ± 13.0	392.6 ± 14.2
Low	173.4 ± 7.4	259.5 ± 8.6	317.6 ± 10.9	367.0 ± 13.3	399.1 ± 17.1	170.9 ± 13.6	230.6 ± 16.1	294.4 ± 19.8	344.0 ± 24.3	382.0 ± 30.4
Middle	171.8 ± 9.8	256.2 ± 13.9	309.9 ± 16.1	356.6 ± 18.3	390.3 ± 19.7	170.6 ± 13.2	226.0 ± 18.0	285.5 ± 24.4	334.4 ± 29.3	370.1 ± 33.6
High	172.5 ± 9.0	253.3 ± 12.3	310.5 ± 15.5	357.9 ± 18.4	390.6 ± 24.0	171.0 ± 13.1	231.7 ± 18.7	293.4 ± 24.3	344.7 ± 27.1	384.8 ± 33.8

Data were presented as means ± SD (n = 5, per group for male and female rats, after 28-day administration). *$P < 0.05$ vs. the control group. [a] Control: equal volume of sterile water; [b] NRICM101 or 102, Low: 1.6 g/kg bw/day; Middle: 3.2 g/kg bw/day; High: 4.8 g/kg bw/day.

Table S4. Effect of NRICM101 or 102 on body weight change in the repeated subacute oral toxicity test.

Body weight change (%)								
Sample	NRICM101				NRICM102			
Week	1	2	3	4	1	2	3	4
Female								
Control[a]	17.2 ± 3.2	15.5 ± 3.5	9.9 ± 2.2	4.0 ± 2.2	25.7 ± 3.4	11.1 ± 4.0	10.1 ± 2.2	4.7 ± 2.1
Low[b]	16.1 ± 4.1	13.6 ± 3.3	9.4 ± 2.2	5.5 ± 1.9	28.7 ± 3.9	10.8 ± 2.7	6.3 ± 2.6*	6.6 ± 1.3
Middle	18.4 ± 1.8	12.0 ± 3.3	9.0 ± 3.5	6.1 ± 2.4	24.0 ± 4.5	11.0 ± 2.7	7.3 ± 3.7	5.8 ± 3.4
High	20.0 ± 3.7	14.1 ± 3.3	8.2 ± 2.8	5.5 ± 2.2	25.3 ± 3.8	11.2 ± 4.4	8.6 ± 4.4	4.5 ± 3.0
Male								
Control	33.5 ± 3.3	29.3 ± 2.0	19.3 ± 2.7	12.0 ± 1.7	49.0 ± 4.1	23.8 ± 3.0	16.2 ± 3.1	9.5 ± 1.8
Low	35.1 ± 3.8	27.7 ± 3.4	16.9 ± 4.8	11.0 ± 3.6	49.7 ± 3.3	22.4 ± 1.4	15.5 ± 1.4	8.7 ± 1.4
Middle	32.5 ± 2.5	26.3 ± 2.0*	17.1 ± 1.5	10.7 ± 2.1	49.2 ± 4.9	21.0 ± 2.9*	15.1 ± 1.8	9.4 ± 1.5
High	35.6 ± 4.8	26.6 ± 2.0*	17.6 ± 1.7	11.6 ± 2.2	46.9 ± 3.6	22.6 ± 1.6	15.3 ± 0.9	9.1 ± 1.8

Body weight change (%) =[BW of week #n - BW of week #(n-1)]/[BW of week#(n-1)]*100%; Data were presented as means ± SD (n = 10, per group for male and female rats, after 28-day administration). *$P < 0.05$ vs. the control group. [a] Control: equal volume of sterile water; [b] NRICM101 or 102, Low: 1.6 g/kg bw/day; Middle: 3.2 g/kg bw/day; High: 4.8 g/kg bw/day.

Table S5. Effect of NRICM101 or 102 on food consumption in the repeated subacute oral toxicity test.

Food consumption (g)								
Sample	NRICM101				NRICM102			
Week	1	2	3	4	1	2	3	4
Female								
Control[a]	18.1 ±0.7	18.8 ± 0.4	19.0 ± 0.6	17.9 ± 0.6	18.6 ± 1.0	18.9 ± 0.9	19.6 ± 1.1	19.8 ± 1.3
Low[b]	17.0 ± 0.8*	17.2 ± 0.8*	18.1 ± 0.8	17.3 ± 0.7	18.2 ± 0.8	18.2 ± 0.8	18.7 ± 0.8	18.8 ± 0.9
Middle	17.8 ± 0.4	17.5 ± 0.8*	18.1 ± 0.6	17.7 ± 0.5	17.4 ± 0.7	17.5 ± 0.8	17.5 ± 0.7 *	18.0 ± 0.8 *
High	17.8 ± 0.4	17.5 ± 0.8*	18.1 ± 0.6	17.7 ± 0.5	18.3 ± 1.1	18.5 ± 1.2	18.4 ± 1.3	18.4 ± 1.4
Male								
Control	24.4 ± 0.9	26.8 ± 0.9	29.4 ± 1.0	29.1 ± 1.4	26.2 ± 0.9	28.4 ± 2.0	30.5 ± 2.9	31.4 ± 2.8
Low	24.4 ± 0.9	26.1 ± 1.4	28.6 ± 1.4	28.4 ± 1.5	26.2 ± 0.8	28.5 ± 1.1	29.6 ± 1.1	29.0 ± 0.8
Middle	23.4 ± 0.8	25.2 ± 1.0	27.3 ± 1.1*	26.9 ± 1.1	26.0 ± 0.8	28.4 ± 0.7	29.0 ± 1.1	29.1 ± 1.1
High	24.1 ± 0.7	26.2 ± 1.1	28.8 ± 1.2	28.1 ± 1.4	25.1 ± 1.4	27.2 ± 1.3	27.9 ± 1.7	28.3 ± 1.7 *

Data were presented as means ± SD (n = 10, per group for male and female rats, after 28-day administration). *$P < 0.05$ vs. the control group. [a] Control: equal volume of sterile water; [b] NRICM101 or 102, Low: 1.6 g/kg bw/day; Middle: 3.2 g/kg bw/day; High: 4.8 g/kg bw/day.

Table S6. The mutagenicity of NRICM101 or 102 in *S. typhimurium* strains in the absence or presence of S9 mix.

NRICM101 (mg/plate)[a]	TA97a	TA98	TA100	TA102	TA1535	NRICM102 (mg/plate)[a]	TA97a	TA98	TA100	TA102	TA1535
Without S9						Without S9					
5	152.7 ± 11.2	29.0 ± 2.0	158.7 ± 9.5	407.0 ± 17.1	14.3 ± 3.8	5	127.7 ± 14.3	18.0 ± 4.4	137.0 ± 3.6	413.0 ± 9.6	12.0 ± 2.0
2.5	120.3 ± 11.8	25.7 ± 3.2	152.0 ± 5.3	425.3 ± 19.1	14.3 ± 6.7	2.5	121.0 ± 6.6	21.7 ± 1.5	122.3 ± 14.7	464.0 ± 12.3	15.7 ± 0.6
1.25	131.3 ± 2.5	28.7 ± 5.1	132.0 ± 15.5	405.3 ± 16.8	11.7 ± 2.1	1.25	110.7 ± 6.7	24.3 ± 3.5	137.3 ± 5.1	446.7 ± 18.0	13.3 ± 3.5
0.625	125.3 ± 3.5	28.0 ± 2.6	121.7 ± 11.0	413.7 ± 20.6	15.0 ± 1.0	0.625	121.0 ± 7.9	24.3 ± 6.0	115.7 ± 4.0	430.3 ± 16.3	14.3 ± 2.5
0.3125	132.0 ± 4.0	28.7 ± 2.1	127.7 ± 12.6	428.0 ± 19.1	12.3 ± 1.2	0.3125	117.7 ± 6.0	24.3 ± 2.3	129.7 ± 3.1	425.3 ± 10.8	15.7 ± 2.5
NC[b]	121.7 ± 8.6	26.3 ± 3.5	135.3 ± 10.4	429.7 ± 19.1	13.3 ± 3.1	NC[b]	131.3 ± 6.1	22.7 ± 6.0	115.7 ± 10.3	409.0 ± 9.5	15.7 ± 3.5
PC[c]	481.0 ± 30.3	701.0 ± 85.8	360.3 ± 18.6	1599.7 ± 92.6	256.7 ± 42.1	PC[c]	499.7 ± 17.9	629.3 ± 72.9	400.0 ± 49.8	1553.3 ± 53.5	307.0 ± 77.1
With S9						With S9					
5	173.3 ± 3.5	39.3 ± 2.9	151.0 ± 9.2	415 ± 11.5	19.3 ± 2.5	5	123.0 ± 4.4	20.3 ± 2.3	142.3 ± 11.0	469.3 ± 10.0	18.7 ± 3.5
2.5	157.0 ± 1.0	41.3 ± 4.7	124.3 ± 8.1	405.3 ± 8.1	16.0 ± 3.0	2.5	147.7 ± 10.0	31.7 ± 1.5	142.3 ± 8.1	496.3 ± 19.8	19.0 ± 2.6
1.25	142.3 ± 4.9	35.0 ± 7.8	135.3 ± 7.0	432.7 ± 23.8	14.7 ± 4.7	1.25	145.7 ± 11.9	29.7 ± 3.2	165.0 ± 5.0	478.0 ± 45.2	13.3 ± 0.6
0.625	151.0 ± 7.8	34.3 ± 3.5	163.3 ± 3.5	426.7 ± 6.7	14.7 ± 4.2	0.625	142.7 ± 8.7	24.7 ± 0.6	149.0 ± 4.6	444.0 ± 23.5	13.0 ± 5.2
0.3125	142.3 ± 12.0	36.7 ± 3.5	140.7 ± 11.0	431.7 ± 11.0	16.0 ± 4.6	0.3125	142.0 ± 5.3	28.7 ± 2.5	141.7 ± 8.0	478.0 ± 12.1	17.0 ± 2.6
NC[b]	143.3 ± 4.0	31.0 ± 8.9	138.7 ± 8.1	466.0 ± 11.5	13.3 ± 2.5	NC[b]	134.7 ± 7.4	26.0 ± 1.0	136.3 ± 3.5	435.0 ± 21.1	16.7 ± 3.5
PC[d]	501.0 ±37.0	305.0 ± 78.6	355.3 ± 55.8	1073.0 ± 43.3	146.3 ± 30.7	PC[d]	510.0± 27.6	264.3 ± 34.0	340.0 ± 40.3	1083.3 ± 104.7	168.0 ± 23.3

[a] NRICM101 or 102 was dissolved in sterile water; [b] Negative control (NC): sterile water; [c] Positive controls (PC): 10.0 µg/plate of 4-nitro-*o*-phenylenediamine (NPD) for TA97a and TA98; 0.4 µg/plate of sodium azide (SA) for TA100 and TA1535; 0.5 µg/plate of mitomycin C (MMC) for TA102.; [d] Positive controls (PC): 4.0 µg/plate of 2-aminofluorene (2-AF) for TA97a and TA100; 4.0 µg/plate of benzo[*α*]pyrene (BP) for TA98; 4.0 µg/plate of 2-aminoanthracene (2-AA) for TA102 and TA1535. Data were presented as means ± SD (n = 3). *$P < 0.05$ vs. the control group.

Table S7. The toxicity and mutagenicity of NRICM101 or 102 in L5178YTk+/- cells with/without S9 fraction by the mouse lymphoma thymidine-kinase assay (MLA).

NRICM101 (mg/ml) [a]	Viability (%)	Non-selective Medium (2 cells/well)	Selective Medium (TFT) (2000 cells/well)	M.F. (10^{-6})	NRICM102 (mg/ml) [a]	Viability (%)	Non-selective Medium (2 cells/well)	Selective Medium (TFT) (2000 cells/well)	M.F. (10^{-6})
		P.E. %[e]	C.E. % (10^{-4})				P.E. %	C.E. % (10^{-4})	
Without S9					Without S9				
5	93.8 ± 3.2	52.8 ±7.4	121.5 ± 20.4	229.9 ± 10.8	5	89.9 ± 7.0	62.3 ± 4.6	103.8 ± 6.4	166.7 ± 2.4
2.5	98.4 ± 0.8	112.4 ± 12.2	177.7 ± 23.6	157.8 ±7.4	2.5	95.7 ± 0.6	101.7 ± 8.0	106.1 ± 16.3	104 ± 8.6
1.25	99.4 ± 0.6	111.5 ± 2.8	139.4 ± 17.4	124.9 ± 13.3	1.25	95.3 ± 4.1	117.0 ± 8.1	95.5 ± 15.8	81.3 ± 8.0
NC[b]	96.8 ± 1.5	101.5 ± 6.2	82.9 ± 7.1	81.6 ± 2.7	NC[b]	97.4 ± 1.8	125.0 ± 10.3	76.8 ± 3.5	61.6 ± 2.4
PC[c]		98.8 ± 4.4	524.3 ± 22.9	530.5 ± 7.8	PC[c]		110.0 ± 5.3	382.0 ± 6.5	348.1 ± 14.9
With S9					With S9				
5	91.1 ± 6.4	89.1 ± 9.4	106.1 ± 13.3	119.2 ± 11.6	5	96.4 ± 2.2	148.4 ± 10.2	72.9 ± 12.1	48.9 ± 4.8
2.5	91.5 ± 3.5	122.8 ± 9.0	99.7 ± 13.2	81.0 ± 5.1	2.5	91.7 ± 2.7	148.4 ± 10.2	63.1 ± 20.8	42.0 ± 11.1
1.25	93.2 ± 2.5	120.8 ± 9.2	101.8 ± 13.4	84.1 ± 4.6	1.25	95.1 ± 1.1	142.8 ± 14.5	82.9 ± 3.5	58.3 ± 4.2
NC[b]	93.0 ± 1.4	84.0 ± 7.1	85.0 ± 6.2	101.2 ± 2.8	NC[b]	97.9 ± 0.7	144.7 ± 5.3	68.8 ± 6.9	47.5 ± 3.1
PC[d]		52.3 ± 7.4	275.6 ± 10.5	532.8 ± 60.0	PC[d]		69.9 ± 9.4	232.5 ± 20.8	334 ± 15.9

[a] NRICM101 or 102 was dissolved in 10% D-PBS; [b] Negative control (NC): D-PBS; [c] Positive control (PC): EMS (0.32 mg/mL); [d] Positive control (PC): 2-AAF (0.2 mg/mL); [e] Plating efficiency (P.E.%); Cloning efficiency (C.E.%); Mutation frequency(MF). Data were presented as means ± SD (n = 3). *$P < 0.05$ vs. the control group.

Table S8. Effects of NRICM101 or 102 on the percentages of reticulocytes and the frequencies of micronucleated reticulocytes in ICR mice.

Treatment (mg/kg bw)		NRICM101		NRICM102	
		Number of micronucleated reticulocytes (MN/5,000 RET, %)	Number of reticulocytes (RET/5,000 RBC, %)	Number of micronucleated reticulocytes (MN/5,000 RET, %)	Number of reticulocytes (RET/5,000 RBC, %)
2.25	male	0.35 ± 0.06	1.40 ± 0.20	0.67 ± 0.27	1.50 ± 0.14
4.50	male	0.39 ± 0.15	1.43 ± 0.57	0.53 ± 0.32	1.46 ± 0.40
6.75	male	0.44 ± 0.21	1.36 ± 0.37	0.51 ± 0.15	1.38 ± 0.36
	female	0.46 ± 0.18	1.27 ± 0.43	0.59 ± 0.35	1.58 ± 0.44
NC[a]	male	0.41 ± 0.13	1.28 ± 0.27	0.54 ± 0.24	1.81 ± 0.36
PC[b]	male	9.16 ± 6.67*	0.03 ± 0.01*	9.98 ± 1.69*	0.02 ± 0.01*

Data were presented as means ± SD (n = 5). *$P < 0.05$ vs. Negaitivecontrol group using Dunnett's test. [a] Negative control (NC): equal volume of sterile water; [b] Positive control (PC): 100 mg/kg bw of cyclophosphamide; Abbreviations: RET, reticulocyte; RBC, red blood cell; MN, micronucleus.
